# Predictive mixed-gas detection using rGO/In_2_O_3_ nanocomposite sensors assisted by machine learning

**DOI:** 10.1039/d5na01092f

**Published:** 2026-02-02

**Authors:** Tanya Sood, Saikat Chattopadhyay, P. Poornesh

**Affiliations:** a Manipal Institute of Technology, Manipal Academy of Higher Education Manipal India poornesh.p@manipal.edu poorneshp@gmail.com; b Department of Physics, School of Basic Sciences, Manipal University Jaipur Jaipur 303007 India

## Abstract

Selectivity towards specific analytes and detection at sub-ppm levels remain significant challenges for chemiresistive gas sensors. Hybrid materials, like reduced graphene oxide (rGO) combined with metal oxides, possess higher sensitivity at ultralow concentrations. In this work, rGO/In_2_O_3_ nanocomposite thin films were prepared by incorporating rGO synthesized *via* a modified Hummers' method into nanocrystalline In_2_O_3_, followed by spin coating and post-deposition annealing. Structural characterization confirmed the formation of phase-pure cubic bixbyite In_2_O_3_ with uniform rGO incorporation, providing abundant defect sites and efficient conductive pathways. The optimised rGO/In_2_O_3_ sensor exhibited good stability towards H_2_S with a detection limit as low as 100 ppb. Nevertheless, accurate identification and concentration estimation of target gases in mixed environments remain challenging. To address this, a machine-intelligent framework was employed for simultaneous gas identification and concentration prediction using a single sensor. Features derived from the dynamic response curves allow the classifier to clearly distinguish gas clusters with 99.7% accuracy and correctly predict previously unseen H_2_S, NH_3_, and CO concentrations under interfering conditions. This combined platform opens the door to smart, ultra-low-level gas sensing in real-world, complicated environments, expanding environmental and health monitoring applications.

## Abstract 2

An advanced rGO/In_2_O_3_ nanocomposite sensor integrated with machine learning and PCA enables ultra-sensitive and selective toxic gas detection in complex mixed environments.

## Graphical abstract

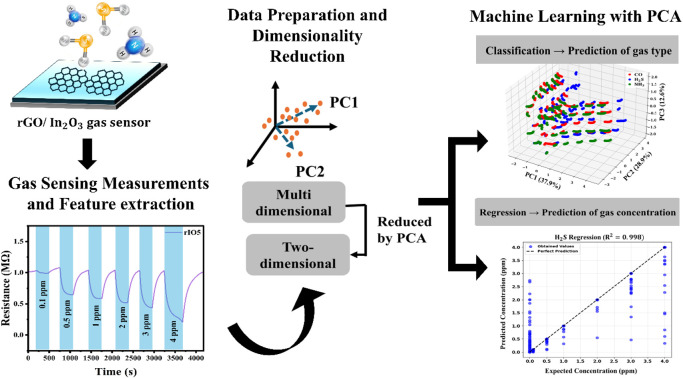

## 1. Introduction

Toxic gas detection has become even more crucial with increasing environmental pollution induced by rapid urbanization and industrialization.^1^ Beyond environmental monitoring, gas sensing plays a key role in fields such as healthcare, food quality control, agriculture, and medical diagnostics. In each of these areas, accurate gas detection is critical for ensuring safety, maintaining quality, or enabling effective diagnostics.^2^ Over these years, metal oxide semiconductors (MO_*x*_) have emerged as leading gas-sensing materials, with device architectures evolving from thick and thin films to nanostructured materials.^3,4^ MO_*x*_ nanostructures possess great sensitivity to a wide range of gases. However, MO_*x*_-based sensors still encounter drawbacks inherent to them.^5^ The general drawbacks include cross-sensitivity to several gases, long-term instability from surface contamination and material degradation, sluggish response and recovery at low concentrations, and low sensitivity at sub-ppm levels.^6^

Various approaches have been studied to overcome these limitations, including catalytic decoration with noble metal nanoparticles, hybrid nanostructures, and microheater integrated devices.^7,8^ Of these, the combination of graphene with MO_*x*_ has some specific advantages.^9^ The high conductivity and low dimensionality of graphene and the surface reactivity of MO_*x*_ create a synergistic effect that leads to an increase in charge transfer, thus enhancing the sensor response.^10^ Reduced graphene oxide (rGO) in specific is a great transducer platform, sensitively replicating interfacial charge interactions on MO_*x*_ surfaces with analyte molecules.^11^ Improvement in the sol–gel synthesis and thin-film deposition methods has also facilitated reliable rGO/MO_*x*_ hybrid sensing layer fabrication with controlled thickness and uniformity.^12^

Despite these advances, the long-standing impediment to practical deployment of gas sensors is the lack of selectivity in complex gas mixtures.^13^ In real-world gas sensing applications, such as industrial safety monitoring, indoor air quality assessment, and environmental surveillance, sensors are routinely exposed to multiple gases rather than single, isolated analytes.^14^ The presence of various interfering species leads to overlapping response signals, making reliable gas identification and concentration estimation extremely challenging for conventional chemiresistive sensors. While sensor arrays and electronic nose systems have been explored to improve selectivity, their dependence on multiple sensing elements significantly increases system complexity, cost, power consumption, and calibration requirements, limiting scalability and widespread deployment.^15^ Consequently, there is a growing demand for single-sensor platforms that combine high sensitivity with intelligent data analysis to enable selective and quantitative detection in mixed-gas environments. From an application standpoint, integrating ultrasensitive sensing materials with machine learning-driven pattern recognition offers a practical pathway toward compact, low-cost, and deployable gas sensing systems. Through the extraction of intrinsic patterns from large sensor datasets, ML provides robust gas classification and concentration prediction beyond the human understanding.^16,17^ However, most prior studies have addressed either classification or concentration regression alone, with integrated analysis of both aspects remaining underdeveloped.

In this work, we present the development of an rGO/In_2_O_3_ nanocomposite gas sensor integrated with a principal component analysis (PCA)-assisted ML strategy. In contrast to previous work, our method simultaneously accomplishes gas classification and concentration prediction in two- and three-dimensional PCA spaces, with the benefits of straightforward visual separability in addition to superior predictive performance.^18,19^ Additionally, we systematically optimized the rGO content in the composite to overcome the inherent sensitivity limitations of the MO_*x*_ component, yielding a hybrid sensor with excellent stability and reproducibility even at sub-ppm gas concentrations. This approach advances the design of intelligent gas-sensing systems capable of selective, ultra-low-level detection in complex real-world environments, with far-reaching implications for environmental safety and health monitoring. At a broader level, these advancements in gas sensing directly support the United Nations Sustainable Development Goals by helping protect occupational and public health (SDG 3) and by enhancing industrial safety, fostering smart sensing innovation, and enabling IoT-based environmental monitoring (SDG 9).

## 2. Experimental details

### 2.1. Preparation of rGO/In_2_O_3_ nanocomposite thin films

Natural graphite powder (purity ≥ 99.9%; particle size ≤500 mesh), potassium permanganate (KMnO_4_), sulfuric acid (H_2_SO_4_), hydrogen peroxide (H_2_O_2_), hydrochloric acid (HCl), ethanol (99.9% purity, C_2_H_5_OH), acetone (CH_3_COCH_3_), isopropanol (C_3_H_8_O), 2-methoxyethanol (C_3_H_8_O_2_), and indium(iii) nitrate hydrate [In(NO_3_)_3_.xH_2_O] (99.99% pure) were procured from Merck Life Science Pvt. Ltd, India. Every chemical used was of analytical grade and employed directly without undergoing any additional purification.

**Fig. 1:**
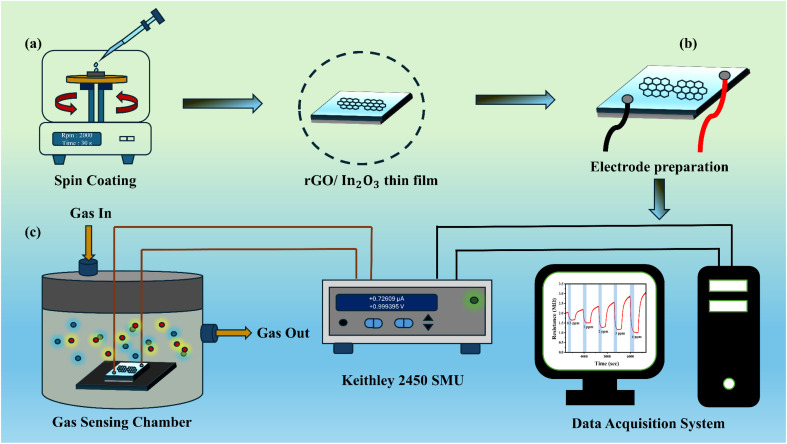
Schematic representation of (a) spin-coated rGO/In_2_O_3_ thin film and (b) electrode preparation and (c) gas sensing setup.

GO powder was synthesized *via* a modified Hummers' method using natural graphite powder.^20^ The resulting GO was washed with DI water and dried in a hot-air oven at 60 °C for 24 h. Glass substrates were cleaned through a series of ultrasonic treatments in laboratory-grade cleaning solution, deionized water (DI), isopropyl alcohol (IPA), and acetone, each for 10 min. The substrates were then dried under a nitrogen flow. To eliminate residual organics and smoothen the surface, an additional 15 min ozone treatment was applied, resulting in cleaner and more uniform substrates. A stable graphene oxide (GO) dispersion was prepared by sonicating the GO powder in ethanol. Separately, a 0.2 M precursor solution in aqueous medium was formulated by dissolving indium nitrate hydrate in 2-methoxyethanol, followed by continuous stirring at 60 °C for 24 h to yield a clear and homogeneous solution. The GO suspension was then added to the In_2_O_3_ precursor solution and sonicated for 2 h to form mixed colloidal dispersions with GO-to-In_2_O_3_ weight ratios of 1, 3, 5 and 7 wt% (hereafter referred to as rIO1, rIO3, rIO5, and rIO7, respectively).The resulting dispersions were deposited onto glass substrates *via* spin coating at 2000 rpm for 30 s. The coated films were heated at 250 °C for 2 min to remove the solvent. Finally, the films were annealed at 350 °C for 2 h in a muffle furnace. For comparison, a pure In_2_O_3_ film (denoted as rIO0) was prepared under identical conditions, excluding the addition of GO. A schematic illustration of the synthesis and fabrication process is shown in Fig. 1(a).

### 2.2. Characterization of rGO/In_2_O_3_ nanocomposite thin films

#### 2.2.1. Structural studies

The crystal structure of the synthesized films was investigated using a Rigaku SMARTLAB X-ray diffractometer with CuKα radiation with a wavelength of 1.5406 Å over a 2*θ* range of 15°–65° at a scanning rate of 1° min^−1^. Raman spectroscopy (Horiba LabRAM HR Evolution) using a 532 nm laser at room temperature was employed to examine the composition, phases, impurities, and structural defects of the films. X-ray photoelectron spectroscopy (XPS) was conducted on an AXIS Supra system with a monochromatic Al Kα source at 14 kV to determine the oxidation states of the constituent elements.

#### 2.2.2. Morphological and optical studies

The morphology and surface features of the synthesized thin films were characterized using field emission scanning electron microscopy (FESEM, JEOL JSM-7610F Plus) operated at 5 kV. Prior to imaging, a thin layer of gold was sputtered onto the samples to enhance the conductivity. UV-visible transmission spectra of the films were recorded using a 1900i UV-vis spectrophotometer in the wavelength range of 190–1100 nm, with bare glass substrates used as the reference. The resulting spectra are presented in Fig. S2 (SI). Photoluminescence (PL) measurements were carried out utilizing a JASCO FP-8300 spectrofluorometer at room temperature, with excitation at 335 nm, and the emission spectra (350–700 nm) are shown in Fig. S3 (SI).

#### 2.2.3. Gas sensing measurements

To enable ohmic contact for gas sensing measurements, a homogeneous conductive silver paste layer was applied to the sensor surface, defining the individual electrode area as 0.5 cm × 0.5 cm (Fig. 1(b)). The sensor was positioned within an airtight stainless-steel chamber fitted with magnetic probes that made direct contact with the silver-coated electrodes. An external heating unit was used to gradually elevate the sensor to its optimal operating temperature. Prior to gas exposure, high-purity synthetic air (comprising 20.9% O_2_ and remaining N_2_, 99.99% purity) was uninterruptedly introduced into the chamber to regulate the sensor's baseline resistance. The total gas flow ratio was regulated at 500 sccm with accurate mass flow controllers (MFCs, Alicat Scientific). Target gas concentrations in the range of 0.1–4 ppm were generated by serial dilution of certified standard gas cylinders (Chemix Specialty Gases and Equipment) with synthetic air, while nitrogen was used solely as the carrier gas for dilution purposes.

During gas sensing measurements, the relative humidity inside the sealed chamber was continuously monitored using a calibrated humidity sensor (DHT11, ±5% RH). Although the initial ambient humidity inside the chamber was approximately 73%, it progressively decreased and stabilized at ∼25% during sensor operation at 250 °C due to thermally induced water desorption. Therefore, the reported humidity corresponds to the stabilized operating condition rather than an externally controlled parameter. Real-time resistance variations during gas exposure and recovery were recorded using a Keithley 2450 Source Meter, and the experimental configuration is illustrated in (Fig. 1(c)). Gas sensing properties were evaluated according to response time, recovery time, and relative sensor response. The response time (*τ*_res_) was the time required for the sensor to reach 90% of its highest resistance while exposing the sensor to a gas, while recovery time (*τ*_rec_) was the time taken by the sensor to recover to 90% of its initial resistance after removal of the test gas and resupply with synthetic air. The sensor responses were calculated by using equations *S*% = ((*R*_a_ – *R*_g_)/*R*_a_) × 100 for reducing gases and *S*% = ((*R*_g_ − *R*_a_)/*R*_a_) × 100 for oxidizing gases, where *R*_a_ and *R*_g_ are the resistance of the sensor in air and in the presence of target gas, respectively.^21^ Each measurement was done in three consecutive cycles to verify reproducibility, with the values presented as mean ± standard deviation. The error bars on the graphs represent the standard deviation of repeated measurements.

##### 2.2.3.1. Data preprocessing

Nine features were extracted from the dynamic response curves for machine learning analysis, as defined in Table S14 (SI). These high-dimensional data were mapped to two- and three-dimensional spaces by principal component analysis (PCA), so that the performance of classification and regression could be visualised clearly. Signal processing, feature extraction, classification, and visualization were performed using Python and Origin.

For regression analysis, the processed dataset was partitioned into training (56%), validation (14%), and test (30%) subsets using stratified random sampling to ensure proportional representation of each gas type (CO, NH_3_, and H_2_S) and concentration range across all subsets. All preprocessing operations, including imputation, scaling, and encoding, were implemented using parameters learned exclusively from the training data to avoid information leakage.

While the raw sensor response is inherently time-dependent, each feature vector corresponds to a characteristic representation of a gas exposure event and is therefore treated as an independent observation for concentration regression. Stratified random splitting thus provides a robust estimate of generalization across different gas types and concentration ranges; however, future work will explore strict temporal hold-out strategies to explicitly account for time-series dependencies under continuous real-time sensing conditions.

## 3. Results and discussion

### 3.1. Structural investigations

#### 3.1.1. XRD analysis

**Fig. 2:**
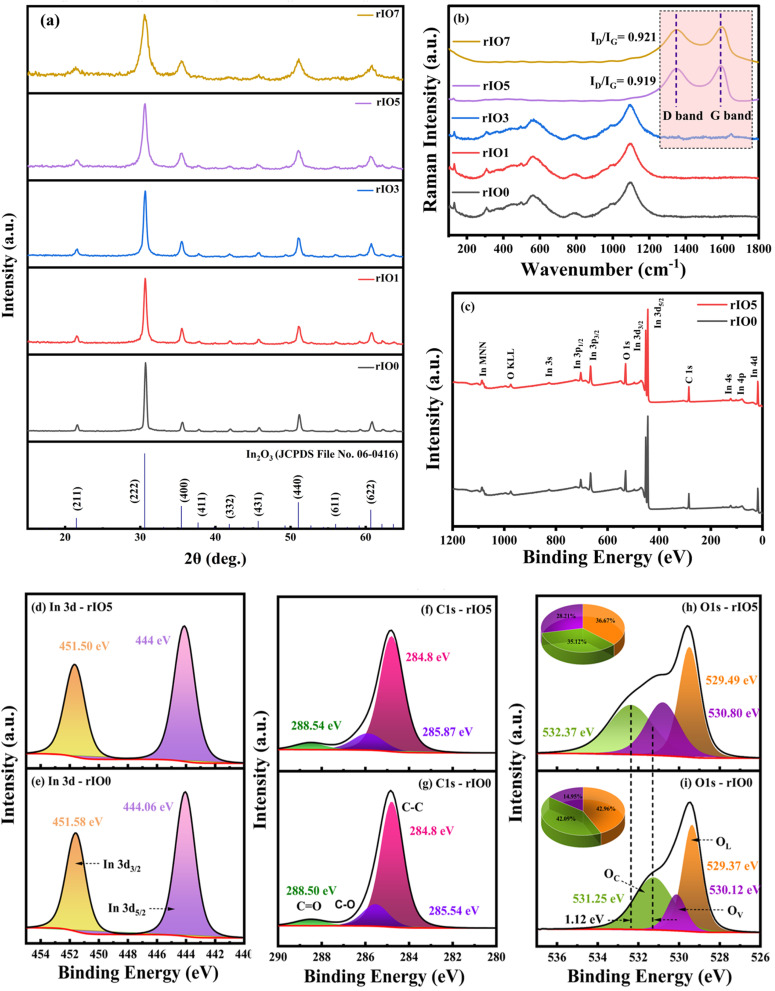
(a) XRD patterns and (b) Raman spectra of rIO0 and rGO/In_2_O_3_ nanocomposites. (c) XPS survey spectra of rIO0 and rIO5. High-resolution XPS spectra of (d and e) In 3d, (f and g) C 1s, and (h and i) O 1s core levels for rIO5 and rIO0, respectively.

The XRD diffractograms (Fig. 2(a)) of all the deposited rIO0 and rGO/In_2_O_3_ composite films exhibit typical diffraction patterns for polycrystalline materials. The major diffraction peak found at around 30.71° is due to the (222) crystal plane, along with several subsidiary peaks that signifies the polycrystalline nature of the deposited films. The identified diffraction peaks are successfully indexed to the body-centered cubic bixbyite structure of In_2_O_3_, consistent with the reference pattern ICDD 06-0416.^22^ The XRD patterns of rGO/In_2_O_3_ composites with different concentrations of rGO retain the same cubic crystallographic phase as rIO0, assuring structural integrity across the composition range investigated. Most significantly, the typical diffraction signature of rGO, usually seen at about 25°, is absent in all composite patterns. This absence can be explained by the comparatively low levels of rGO loading and the weak diffraction intensity inherent to rGO sheets.^23^ Different structural parameters were obtained from XRD patterns using the Williamson–Hall method by applying eqn (S1)–(S5) (SI) and are summarised in Table 1. The calculated parameters indicate systematic microstructural development with the increase in the rGO concentration. A progressive reduction in crystallite size is achieved with increasing rGO content between 0 and 7 wt%, indicating that rGO indeed hinders crystal growth processes and favours the development of smaller crystalline domains.^24^ This crystallite size refinement signifies a substantial advantage for gas sensing applications since smaller grain sizes are usually associated with higher sensitivity, faster response kinetics, and better detection thresholds. Simultaneously, both the lattice strain and dislocation density exhibit marked increases with increasing rGO concentration, indicating that rGO incorporation generates substantial structural imperfections and lattice distortions at the heterointerfaces between the two phases.

**Table 1:** Structural parameters of rIO0 and rGO/In_2_O_3_ nanocomposites calculated using the Williamson–Hall method

rGO concentration (wt%)	2*θ* (deg.)	Crystallite size *D* (nm)	Dislocation density *δ* (×10^15^ lines per m^2^)	Strain *ε* (×10^−3^)
0	30.71	25.90	1.36	0.43
1	30.66	24.96	1.60	1.32
3	30.64	21.10	2.25	0.97
5	30.61	15.81	4.00	2.57
7	30.61	14.86	4.53	7.79

#### 3.1.2. Raman spectroscopic analysis

Raman spectroscopy was used to examine the vibrational characteristics and structural features of rIO0 and the rGO/In_2_O_3_ composites rIO1, rIO3, rIO5, and rIO7, as shown in Fig. 2(b). rIO0 adopts a cubic bixbyite structure with the space group I^3^_a_ (T^7^_h_ in Schoenflies notation). Based on group theory calculations, the predicted vibrational modes for this crystal structure are:^25^

1 Γ = 4A_g_ + 4E_g_ + 14T_g_ + 5A_u_ + 5E_u_ + 16T_u_

In this notation, A_g_, E_g_, and T_g_ modes are Raman-active, T_u_ modes are infrared-active, while A_u_ and E_u_ modes are optically inactive. The experimental Raman spectrum of rIO0 revealed eight distinct peaks. The lowest frequency mode at 129 cm^−1^ arises from In–O bond stretching. The peak at 306 cm^−1^ originates from bending vibrations of InO_6_ octahedra, denoted as *δ*(InO_6_). The peak at 364 cm^−1^ corresponds to In–O–In bridging bonds' stretching vibrations. The three peaks at 453, 491, and 559 cm^−1^ correspond to various InO_6_ octahedral stretching modes, *ν*(InO_6_). Complex vibrational modes and an overtone band are indicted by the higher frequency peaks at 786 cm^−1^ and 1092 cm^−1^, respectively. Precisely, the 1092 cm^−1^ peak is the first overtone of the 559 cm^−1^ fundamental vibration.^26^ With increasing rGO content in the composites, the In_2_O_3_ peak intensities decreased systematically. This decrease indicates the fact that rGO sheets increasingly cover the surfaces of In_2_O_3_ nanoparticles, which diminishes their Raman signals.

The typical carbon signatures only became observable in rIO5 and rIO7. The two strong carbon bands were evident: the D band at 1347 cm^−1^ and the G band at 1590 cm^−1^. The D band indicates structural disorder and defects in the carbon lattice, while the G band signifies well-ordered sp^2^ carbon networks characteristic of graphitic materials.^27^ Determination of the *I*_D_/*I*_G_ intensity ratios provided values of 0.919 and 0.921 for the rIO5 and rIO7 samples, respectively. The ratios measure the extent of structural disorder in carbon materials; greater values imply more extensive defects.^27^ The comparable ratios imply equivalent defect densities in both high-loading samples. The defects are the consequence of the thermal reduction process converting GO to rGO, which will cause structural flaws within the sp^2^ carbon matrix. The systematic variation of spectral features with varying rGO loadings confirms successful integration of the carbon component and exhibits the progression of interfacial interactions between In_2_O_3_ and rGO phases within these composite materials.

#### 3.1.3. XPS investigation

X-ray photoelectron spectroscopy (XPS) was carried out to analyse the elemental composition and chemical states of the nanocomposite films. The survey spectra for rIO0 and rIO5 are shown in Fig. 2(c), with all spectra calibrated against the C 1s peak at 284.4 eV. Distinct photoemission peaks were observed in the spectra corresponding to indium and oxygen states, consisting of In 3s, In 3p, In 3d, In 4s, In 4p, In 4d, and O 1s, accompanied by Auger features assigned to In MNN and O KLL, in addition to the C 1s signal. The observed characteristic binding energies (BE) were 828 eV (In 3s), 703 eV (In 3p_1/2_), 665 eV (In 3p_3/2_), 452 eV (In 3d_3/2_), 444 eV (In 3d_5/2_), 123 eV (In 4s), 78 eV (In 4p), 17 eV (In 4d), and an In MNN peak within 1076–1084 eV.^28^ To gain deeper insights into the chemical environment, high-resolution scans of the In 3d, C 1s, and O 1s core levels were also examined.

##### 3.1.3.1. High-resolution In 3d spectra

Fig. 2(d) and (e) present the Gaussian-deconvoluted In 3d high resolution spectra of rIO5 and rIO0 nanocomposites, respectively. The spectra reveal two characteristic components, In 3d_5/2_ and In 3d_3/2_, with binding energies at 444 eV and 451.5 eV, reflecting the spin–orbit splitting of the In 3d levels.^26^ These peaks prove the presence of trivalent indium ions (In^3+^) in the films. The energy difference between In 3d_5/2_ and In 3d_3/2_ is ∼7.5 eV, in agreement with earlier reported values.^28^

##### 3.1.3.2. High-resolution C 1s spectra

Fig. 2(f) and (g) display high-resolution C 1s spectra for rIO5 and rIO0, respectively. The 284.8 eV peak is associated with carbon atoms in graphitic environments, both sp^2^- and sp^3^-hybridized C–C bonds. The peak around 285.54 eV (which shifts to 285.87 eV in samples containing rGO) is associated with epoxy and alkoxy groups (C–O–H) and represents the presence of oxygenated carbon species.^29^ The peak at 288.50 eV is attributed to carboxyl groups (O–C=O).^30^ The shift of the second peak by 0.33 eV after the addition of rGO indicates a change in the chemical environments of these oxygenated carbons. The shift indicates that the addition of rGO favours better interaction with In_2_O_3_, which can be due to redistribution of charge or enhanced bonding involving oxygen-containing functionalities such as hydroxyls or epoxies.^31^

##### 3.1.3.3. High-resolution O 1s spectra

The high-resolution O 1s spectra shown in Fig. 2(h) and (i) exhibit three well-separated bands, each corresponding to different oxygen species. The band near 529 eV is assigned to lattice oxygen (O_L_), *i.e.*, corresponding to In–O–In bonds. The band at around 530 eV is attributed to oxygen atoms with vacancies (O_V_) or hydroxyl-type functional groups, and the band around 531 eV is assigned to surface-adsorbed oxygen (O_C_).^32^ Quantitative analysis of the peaks areas show that the ratio of oxygen vacancies increases from 14.95% in rIO0 to 28.91% in rIO5 upon addition of rGO. As a result, the oxygen vacancy content is enhanced in the composite.^33^ The addition of rGO facilitates the formation of oxygen vacancies, and this observation agrees with trends reported in complementary analyses like Raman and PL measurements.

### 3.2. Morphology of In_2_O_3_ and rGO/In_2_O_3_ nanocomposites

**Fig. 3:**
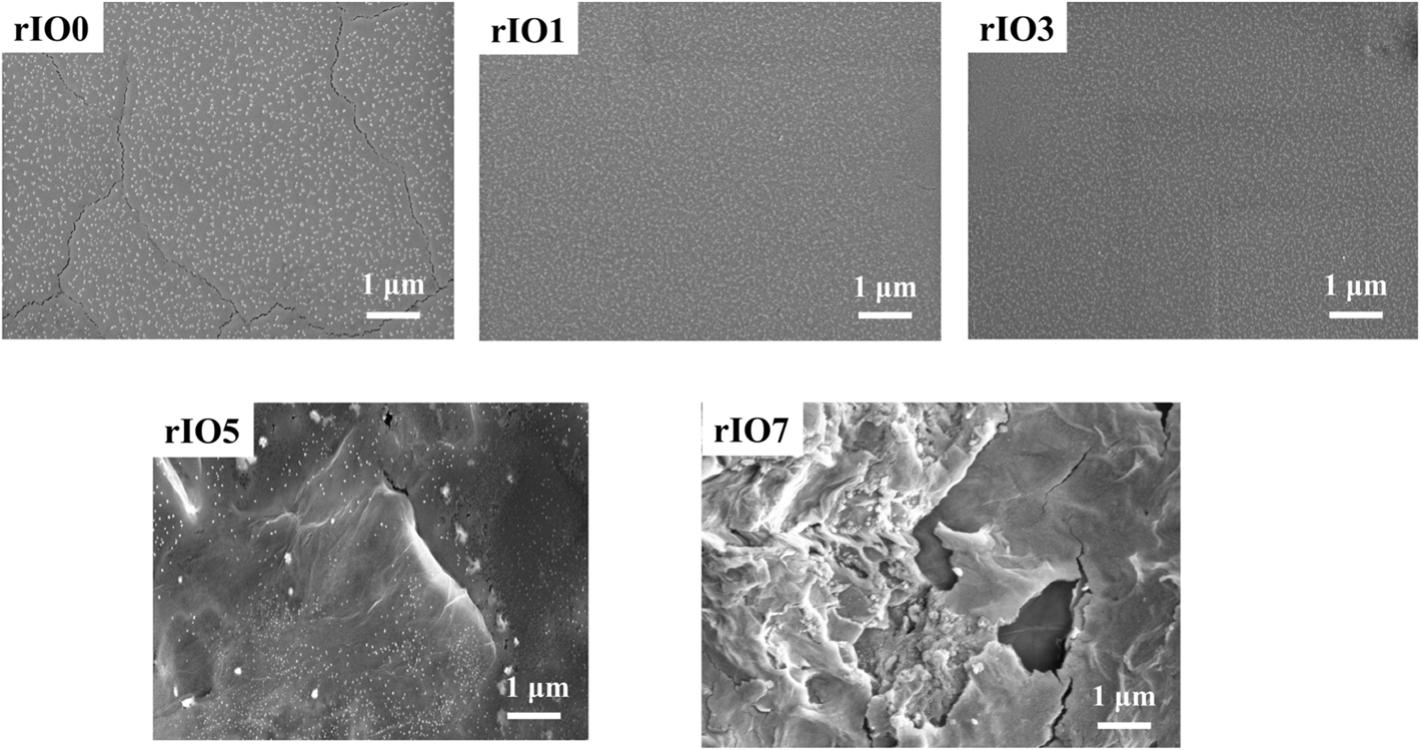
FESEM images of rIO0 and rGO/In_2_O_3_ nanocomposites.

The morphological characteristics of rIO0 and rGO/In_2_O_3_ thin films were analysed using Field Emission Scanning Electron Microscopy (FESEM), and the images are presented in Fig. 3. rIO0 exhibits small, uniformly distributed particles with a fine granular structure. However, micro-cracks are clearly visible on its surface, formed due to shrinkage during annealing. The brittle, granular film cannot accommodate tensile stresses at grain boundaries, resulting in microscopic crack formation and propagation across the surface. In contrast, rGO/In_2_O_3_ composites show complete crack elimination due to the crack bridging capability of rGO sheets.^34^ When cracks attempt to propagate through the In_2_O_3_ matrix, flexible rGO sheets cover the crack openings and provide bridging forces that maintain structural integrity. At lower rGO concentrations (rIO1 and rIO3), the morphology remains like rIO0. The rGO content is barely detectable due to low concentration and the embedding of discrete rGO sheets within hierarchical In_2_O_3_ structures without distinct visible edges.^23^ For rIO5, FESEM micrographs show a crumpled, thin layer that consists of overlapping nanosheets with In_2_O_3_ nanoparticles distributed on the surface. The nanoparticles are mainly attached to rGO surface wrinkles by embedding, indicating successful formation of the rGO/In_2_O_3_ nanocomposite.^35^ The nanoparticles create a porous structure, which will enhance adsorption and is beneficial for gas sensing applications.^36^ For rIO7, more wrinkled rGO structures are seen, with some sheets showing a tendency to aggregate. This aggregation blocks gas diffusion pathways, creating conditions unfavourable for gas-sensing applications.^37^

### 3.3. Gas sensing measurements and data curation

The operating temperature plays a crucial role in determining the sensing performance of metal oxide semiconductor (MOS) gas sensors, as it directly governs gas adsorption, desorption, and surface reaction kinetics. To determine the optimal operating temperature, both rIO0 and rIO5 sensors were systematically evaluated for their response to 4 ppm H_2_S over a temperature range of 150–350 °C, as shown in Fig. S4 (SI). The sensor response increases progressively with temperature from 150 °C to 250 °C due to enhanced activation of surface-adsorbed oxygen species and improved reaction kinetics with H_2_S molecules. At temperatures below 250 °C, the thermal energy is insufficient to overcome the activation barrier required for effective gas–surface interactions, resulting in lower sensor response. Beyond 250 °C, the response decreases because elevated temperatures promote rapid desorption of gas molecules from the sensor surface, reducing the time required for surface reactions.^38^ Therefore, 250 °C represents an optimal balance between adsorption and reaction kinetics, yielding the maximum sensor response. Accordingly, all subsequent gas sensing measurements were performed at this temperature.

**Fig. 4:**
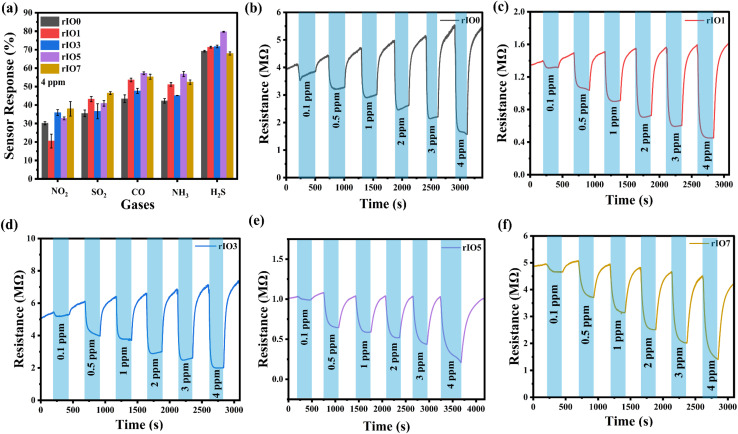
(a) Selectivity histogram of H_2_S, SO_2_, CO, NH_3_ and NO_2_ gases obtained at an operating temperature of 250 °C. Sensor response curves of gas sensors for different rGO concentrations: (b) rIO0, (c) rIO1, (d) rIO3, (e) rIO5, and (f) rIO7, all measured at 250 °C.

In order to determine the selectivity of the In_2_O_3_ and rGO/In_2_O_3_ sensing films, their response toward 4 ppm concentration of NO_2_, SO_2_, CO, NH_3_, and H_2_S was tested at the optimal working temperature of 250 °C,^39^ as shown in Fig. 4(a). The performance showed a considerably stronger response to H_2_S, comparatively moderate responses to NH_3_ and CO, and much weaker responses to SO_2_ and NO_2_. These results emphasize the higher selectivity of the sensors toward H_2_S compared with the other gases tested. As a result, the H_2_S response curves are given in more detail.

**Fig. 5:**
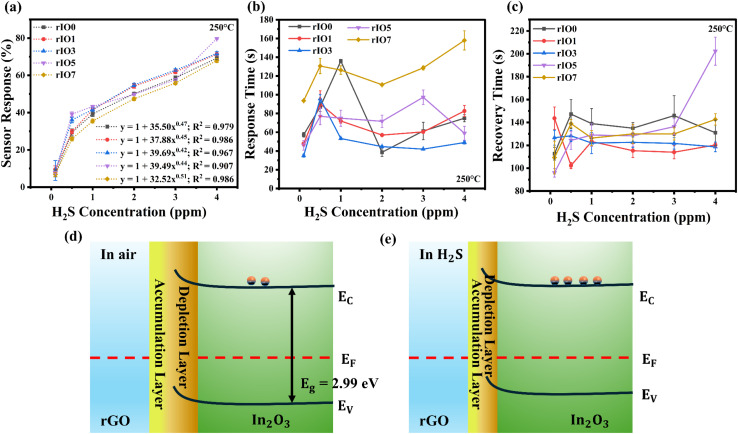
(a) Sensor response, (b) response time, and (c) recovery time *vs.* H_2_S gas concentration for rIO0 and 1, 3, 5, and 7 wt% rGO/In_2_O_3_ nanocomposites. Simplified energy band diagrams of rGO/In_2_O_3_ nanostructures in (d) air and (e) H_2_S.

Fig. 4(b)–(f) show the resistance variation plots of rIO0 and rGO/In_2_O_3_ sensors for H_2_S concentrations ranging from 0.1 ppm to 4 ppm with an operating temperature of 250 °C. When exposed to H_2_S, all sensors showed a decrease in resistance, which later returned to baseline levels after the removal of the gas, attesting to their characteristic n-type semiconducting behavior to a reducing gas.^40^ Notably, the magnitude of resistance change increased with increasing H_2_S concentration. The increase in resistance modulation with increasing H_2_S concentration can be attributed to the enhanced surface redox reactions between H_2_S molecules and chemisorbed oxygen species on the In_2_O_3_ surface. At higher H_2_S concentrations, a larger number of reducing gas molecules react with surface oxygen ions, releasing more electrons back into the conduction band of n-type In_2_O_3_, thereby producing a greater change in resistance as discussed in the sensing mechanism in detail. Furthermore, rGO/In_2_O_3_ composites having up to 5 wt% rGO consistently showed greater resistance variation compared to pure In_2_O_3_ across all tested H_2_S levels. The corresponding sensor responses derived from the resistance data are presented in Fig. 5(a). The H_2_S sensing behaviour of the developed sensors followed a modified power law model expressed as *y* = 1 + *ax*^*b*^, showing strong linearity with high correlation coefficients (*R*^2^ > 0.99).^41^ In this equation, the coefficient *a* is indicative of the rate at which response changes with concentration, effectively representing sensitivity.^42^ The exponent *b*, typically less than 1 for H_2_S, is associated with the surface-level interaction mechanisms between the gas molecules and the sensing material. The gas sensing response demonstrates a composition-dependent trend across the rGO/In_2_O_3_ composite series, exhibiting optimal performance for rIO5 before declining at rIO7 (Table S2). The enhancement in sensor response progresses systematically from rIO0 through intermediate loadings (rIO1 and rIO3), reaching maximum values at rIO5 for multiple gas concentrations, particularly notable with responses of 39.2% at 0.5 ppm, 43.3% at 1 ppm, and 79.7% at 4 ppm. Beyond this optimal threshold, further rGO incorporation into rIO7 results in diminished sensing performance across all tested concentrations, reflecting the complex balance between synergistic enhancement and detrimental overloading effects in the composite system. To estimate the limit of detection (LOD) for each sensor, the adapted power law equations were used with a threshold response value (*y*_min_) of 1.05, based on a signal-to-noise ratio of 3.^43^ Using this approach, the calculated LODs for rIO0, rIO1, rIO3, rIO5 and rIO7 were found to be 56, 48, 43, 45, and 58 ppb, respectively. The improved detection limits and a significantly larger resistance variation compared to pure In_2_O_3_ are due to the synergistic effects of rGO incorporation. The formation of rGO/In_2_O_3_ heterojunctions leads to an expanded electron depletion layer at the interface, which is highly sensitive to surface charge variations induced by gas adsorption. In addition, rGO provides high electrical conductivity, an increased effective surface area, and abundant active sites, facilitating efficient charge transport and enhanced interaction with H_2_S molecules. These combined effects result in a more pronounced modulation of resistance in rGO/In_2_O_3_ composites, particularly at optimized rGO loadings of up to 5 wt%.

The response and recovery times of rIO0 and rGO/In_2_O_3_ sensors, evaluated against H_2_S concentrations ranging from 0.1 to 4 ppm at 250 °C, are illustrated in Fig. 5(b) and (c). It was observed that incorporating rGO into the In_2_O_3_ matrix led to a general reduction in both response and recovery times from rIO0 to rIO5, followed by a slight increase for rIO7. While the rIO5 sensor exhibited the highest sensitivity across the tested concentration range, its response and recovery times were not always the fastest among the series. Notably, the rIO1 and rIO3 sensors demonstrated quicker response times at several concentrations (TS3 and TS4, SI). For instance, at 0.1 ppm H_2_S, the rIO3 sensor achieved a response time of 35 ± 2 s, outperforming both the rIO0 (48 ± 2 s) and rIO5 (46 ± 5 s) sensors. A similar trend was observed at 1 ppm, where the rIO3 sensor responded in just 53 ± 1 s, faster than the rIO1 (72 ± 2 s) and rIO5 (75 ± 9 s) variants, indicating more efficient gas adsorption and reaction kinetics at intermediate rGO levels. The recovery also showed a similar trend. In the lowest concentration tested (0.1 ppm), the rIO5 sensor had the lowest recovery time of 96 ± 4 s, in comparison to 144 ± 10 s and 127 ± 6 s for rIO1 and rIO3 sensors, respectively. But for higher concentrations such as 0.5, 2, and 3 ppm, the rIO1 and rIO3 sensors had slightly more rapid recovery traits. For instance, recovery times for the 2 ppm concentration were 115 ± 6 s (rIO1), 123 ± 5 s (rIO3), and 128 ± 9 s (rIO5). These indicate a compromise between peak sensitivity and dynamic response time, which can be controlled by aspects such as surface energy states, rGO distribution, and diffusion pathways. The sensing performance of rGO/In_2_O_3_ was significantly improved compared to that of the rIO0 sensor.

Furthermore, the stability of the rIO5 sensor was evaluated through both repeatability and long-term stability tests. As shown in Fig. S5(a) (SI), repeatability measurements were performed by repeatedly exposing the sensor to 4 ppm H_2_S at the optimal operating temperature of 250 °C. The sensor response exhibited no significant variation over successive cycles, demonstrating excellent repeatability. In addition, long-term stability (Fig. S5(b)) was assessed by measuring the sensor response to 4 ppm H_2_S at intervals of 5 days. Only minor fluctuations in response were observed over time, indicating good long-term stability of the nanocomposite sensor. These results confirm the reliability and robustness of the rIO5 sensor for sustained gas sensing applications.

The enhanced gas sensing property of rIO5 results from the synergistic enhancement of structural, electronic, and morphological properties attained at the optimum 5 wt% rGO loading.^44^ XRD studies indicate that the rGO addition consistently decreases the crystallite size of In_2_O_3_. The reduced crystalline domains allow improved response kinetics with faster gas diffusion and structural integrity.^45^ Raman spectroscopy ensures that the characteristic In_2_O_3_ vibrations are preserved, though their intensities decrease systematically, signifying successful rGO coverage without active site blockage.^46^ PL and XPS analyses also confirm a remarkable rise in the oxygen vacancy density in rIO5. The ideal vacancy density offers abundant electron donor states with yet sufficient lattice oxygen to ensure baseline resistance stability, hence significantly improving the sensing performance.^47^ FESEM images indicate that rGO also removes surface micro-cracks by its crack-bridging action, which avoids gas leakage *via* defects while ensuring sufficient porosity for surface interaction.^47^ The heterogeneous interface created between rGO and In_2_O_3_ also promotes efficient carrier transport and creates additional active sites for gas adsorption and further enhances the overall sensor enhancement.^27^ The schematic diagram of the rGO/In_2_O_3_ local heterojunction is depicted in Fig. 5(d) and (e), together with a model of interfacial charge distribution. During contact, the electrons move from In_2_O_3_ to rGO until the equilibration of the Fermi level, causing band bending and creating a built-in electric field within the junction.^48^ At the operating temperature, oxygen molecules are adsorbed on the sensor surface and extract electrons from the conduction band, forming chemisorbed oxygen species like O_2_^−^ and O_2_^2−^, which results in the formation of a surface electron depletion layer and an increase in the baseline resistance. The oxygen adsorption processes can be expressed as follows:^49^

2 O_2_(g) + e^−^ ⇌ O_2_^−^(ad)

3 O_2_^−^(ad) + e^−^ ⇌ O_2_^2−^(ad)

Upon exposure to H_2_S, a reducing gas, the gas molecules react with the chemisorbed oxygen species, releasing electrons back into the conduction band. This process reduces the potential barrier height and depletion layer width, facilitating charge transport and resulting in a decrease in sensor resistance. In contrast, oxidizing gases such as NO_2_ extract electrons from the sensing layer, leading to an increase in resistance. The sensing transduction mechanism is therefore based on resistance modulation. The main reactions involved in H_2_S sensing are given as follows:^49^

4 H_2_S(g) + e^−^ ⇌ H_2_S(ad)

5 H_2_S(g) + O_2_^*x*−^(ad) ⇌ H_2_S(ad) + O_2_(g) + *x*e^−^

6 2H_2_S(ad) + 3O_2_^*x*−^(ad) ⇌ 2SO_2_(g) + 2H_2_O + 3*x*e^−^

Importantly, all target gases investigated in this work, namely H_2_S, CO, NH_3_, and SO_2_, are reducing in nature, except NO_2_. These gases interact with the sensor surface through a similar redox-driven mechanism involving reaction with chemisorbed oxygen species or direct electron donation to the sensing layer. Despite the differences in the molecular structure, these gases share the ability to modulate the surface charge density in a comparable manner.^50^

Consequently, the sensor exhibits similar qualitative response characteristics toward different reducing gases, as the resistance change is governed primarily by depletion layer modulation rather than gas-specific chemical binding. While the response magnitude may vary depending on the adsorption strength, reaction kinetics, and gas concentration, the overall response trend remains similar.^51^ This behavior reflects the inherently limited selectivity of a single chemiresistive sensor operating under fixed conditions and highlights the need for strategies such as surface functionalization, catalytic modification, or sensor arrays to achieve improved gas discrimination.

**Fig. 6:**
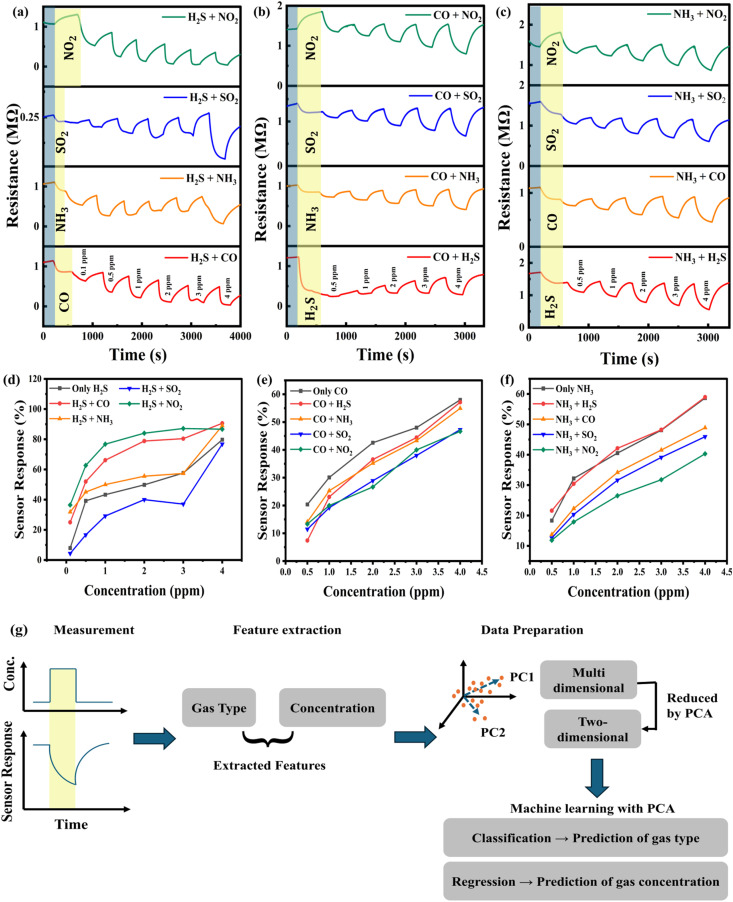
Cross-interference analysis of the rIO5 sensor at an operating temperature of 250 °C. (a) Response to H_2_S (0.1–4 ppm) in the presence of NH_3_, CO, NO_2_, and SO_2_. (b) Response to CO (0.5–4 ppm) under the same conditions. (c) Response to NH_3_ (0.5–4 ppm) under the same conditions. (d–f) Comparison of response values for H_2_S, CO, and NH_3_. (g) PCA-based workflow for gas recognition.

Clearly, rIO5 with higher heterointerface-induced oxygen vacancies was beneficial to the sensing process. Therefore, it was also tested under mixed environment conditions. As previously proven, the rIO5 sensor showed excellent selectivity toward H_2_S. Nevertheless, significant responses were also noticed for NH_3_ and CO, showing possible cross-sensitivity to make it difficult to identify the target gas in mixed environment analytes. To further characterize and quantify the behavior, a series of controlled mixed-gas experiments were aimed at assessing the performance of the sensor in the presence of interfering gases. In the experiments, an interfering gas of fixed concentration was first introduced into the test chamber to establish a background environment. The target gas was then added with the continued background gas flow to mimic a real-life mixed-gas situation. Sensor responses were measured at different concentrations of the target gas, and the obtained data were analysed in order to determine the effect of coexisting species on the detection accuracy. Three typical case studies demonstrate the sensor's cross-interference behavior. In Fig. 6(a) is shown the response curves to the target gas H_2_S, with the introduction of NH_3_, CO, NO_2_, and SO_2_ as interfering species. Fig. 6(b) and (c) show the sensor response to CO and NH_3_ under the same mixed-gas conditions.

Interestingly, the existence of interfering gases not only changed the sensitivity but also influenced the response and recovery dynamics of the rIO5 sensor. For example, in mixed environments, the response of H_2_S was greatly enhanced (*e.g.*, 7.9% for 0.1 ppm H_2_S alone became 25% with CO and 31.8% with NH_3_), and the respective response and recovery times were significantly longer than those under single-gas conditions. At higher concentrations (*e.g.*, 4 ppm H_2_S + NH_3_), both response and recovery times were greater than 270 s, in sharp contrast to ∼60–200 s for H_2_S alone. Comparable aberrations were also experienced by CO and NH_3_ detection under interference from other analytes (Fig. 6(d)–(f)). These extensive datasets are given in the SI (Tables S5–S13). In addition, one cannot identify the target gas in question by simply comparing the response values. This highlights the need for a more comprehensive analysis using statistical and machine learning approaches, such as PCA, ML-based classification, and regression, to accurately recognize gases and estimate their concentrations.^52^

#### 3.3.1. Classification and regression analysis of gases using machine learning

##### 3.3.1.1. Gas classification

Fig. 6(g) presents a schematic of the overall workflow adopted for PCA-based gas recognition. Sensor responses are first recorded for different gas species at varying concentrations. From these response curves, nine characteristic features are extracted (defined in Table S14, SI), and their interdependence is evaluated using a correlation matrix (SI, Fig. S6). These features are then projected into the principal component (PC) space, where two- or three-dimensional scatter plots serve as the machine learning input for gas classification and concentration prediction. This approach integrates dimensionality reduction with machine learning, enabling clear visualization of gas clustering.^48^

The variance distribution of the PCs is shown in SI, Fig. S7a: PC1 and PC2 together explain ∼67% of the total variance, while the first five PCs capture nearly 95%, reducing dimensionality by more than half without major information loss. The feature loadings for the first three PCs (SI, Fig. S7b) further confirm that these components capture the dominant trends in the dataset and reliably represent the overall feature space.

**Fig. 7:**
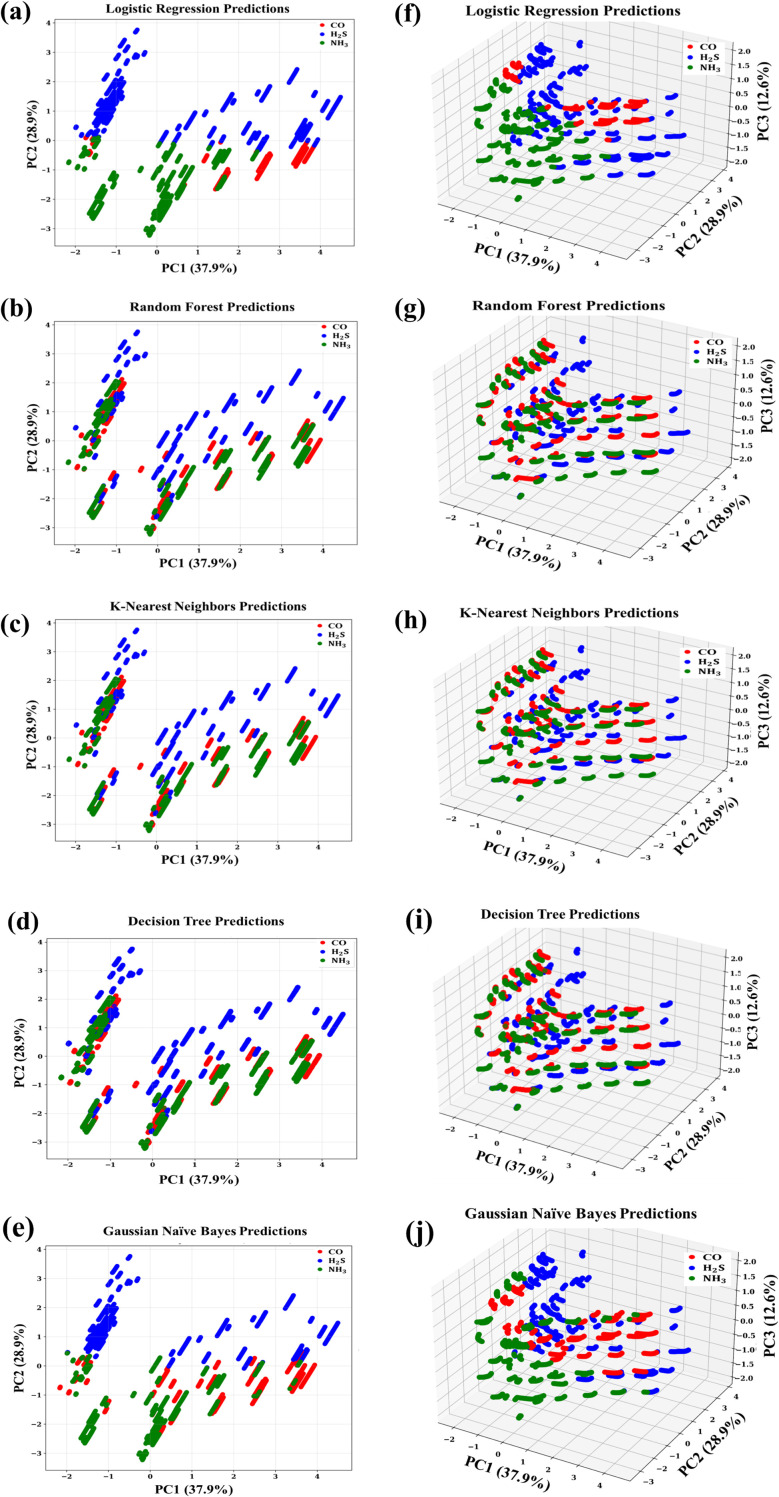
Machine learning-based gas classification in the PCA space. (a–e) 2D projections (PC1 *vs.* PC2, accounting for 37.9% and 28.9% of variance, respectively) for logistic regression, random forest, K-nearest neighbors, decision tree, and Gaussian Naïve Bayes classifiers. (f–j) Corresponding 3D projections including PC3 (12.6%), with CO, H_2_S, and NH_3_ represented in red, blue, and green, respectively.

To verify the suggested scheme for target gas discrimination in binary mixtures, we utilized a supervised machine learning (ML) method, using the principal components (PCs) extracted as feature inputs. Classification was done using standard algorithms, such as logistic regression, K-nearest neighbors (KNN), random forest (RF), Gaussian Naïve Bayes (NB), and decision tree, with respective 2D and 3D PCA scatter plots shown in Fig. 7. Logistic regression had the lowest performance (overall accuracy, ∼ 49%), with clear misclassification of CO and NH_3_ and only some identification of H_2_S (Fig. 7(a) and (f)). This result is consistent with the poor linear separability seen in the PCA feature space. NB did slightly better (accuracy, ∼ 52%) but again performed poorly in areas where there were overlapping class distributions, as expected with violations of its independence and Gaussian assumptions (Fig. 7(b) and (g)). RF, KNN, and decision tree, on the other hand, performed outstanding classification, with overall accuracies over 99%. These findings verify that neighbourhood-based and non-linear models are appropriate to leverage the structure maintained in the PCA space, while linear models are insufficient to model the gas response patterns' complexity. Moreover, dimensionality reduction to the PC space provides a clear visual discrimination of the gas species, as further validated by the confusion matrices of the tested models.

##### 3.3.1.2. Regression analysis

For regression-based prediction of target gas concentrations in binary mixtures, the data were partitioned into training, validation, and test sets with a ratio of 56 : 14 : 30. Hyperparameter optimization was performed for both K-nearest neighbors (KNN) and random forest regressors to ensure reliable model performance. For KNN, the influence of distance metrics (Euclidean, Manhattan, and Minkowski with *p* = 1 and 2), weighting schemes (uniform and distance-based), and the number of neighbors (1–7) was systematically examined. The performance of the model was evaluated by cross-validation for the training and validation sets, and the best configuration was chosen based on the lowest mean squared error (MSE) and the highest coefficient of determination (*R*^2^). The best configuration for the current datasets was that of the Manhattan distance metric with four nearest neighbors and weighting based on the distance. In the random forest regression case, tuning was done by changing the number of trees (*n* estimators), max depth of trees, minimum samples to consider at a node, minimum leaf samples, max samples, max features to consider at each split, and bootstrap. Cross-validation determined the best parameter setting as 267 trees, maximum depth equal to 50, no limit on feature selection at each split, a minimum of six samples per split, and one sample per leaf with bootstrap on. This setting resulted in stable and reproducible predictions for datasets.

**Fig. 8:**
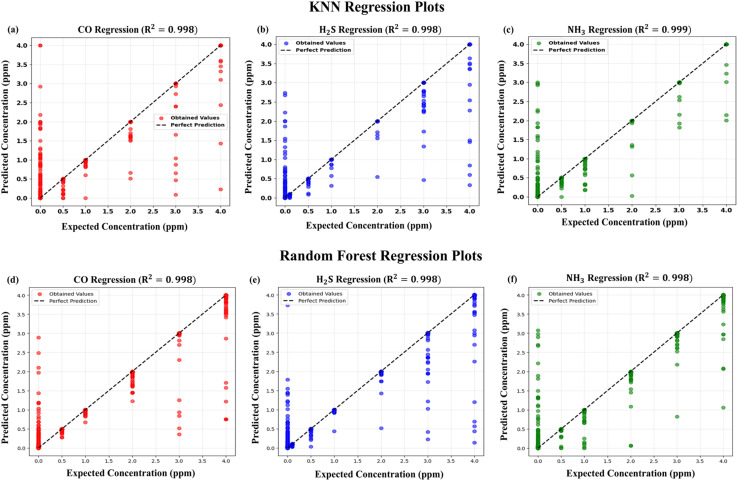
Comparison of KNN and random forest regression models: predicted *vs.* expected concentrations of all the three target gases along the ideal prediction line.

Both models' regression performance is depicted in Fig. 8, where predicted against expected concentrations for CO, H_2_S, and NH_3_ are plotted. Quantitative analysis (Table 2) validated the high predictive efficiency for both regressors, as *R*^2^ values were above 0.997 in all gases. Although random forest provided a slightly inferior RMSE for CO, KNN offered slightly better results for both H_2_S and NH_3_. Both models had extraordinarily low limits of detection (LOD) and quantification (LOQ), as per validation, validating their appropriateness for trace-gas detection. However, random forest tends to be more computationally intensive and can be sensitive to imbalanced datasets, while KNN showed a consistent level of accuracy with less complex parameterization. In summary, these results verify that the PCA-based preprocessing approach not only supports easy visualization of gas classification in a low-dimensional space but also supports precise regression of gas concentrations. This dual functionality supports the promise of PCA-augmented machine learning as a sound framework for pushing qualitative and quantitative gas sensing.

**Table 2:** Performance of K-nearest neighbors and random forest regression models on the mixed-gas dataset

Regression model	Gas name	MAE	MSE	RMSE	NRMSE	*R* ^2^	LOD	LOQ
KNN	CO	0.0025	0.0042	0.0648	1.6190	0.9978	0.2137	0.6476
	H_2_S	0.0015	0.0025	0.0501	1.2526	0.9985	0.1653	0.5010
	NH_3_	0.0015	0.0020	0.0453	1.1313	0.9988	0.1493	0.4525
Random forest	CO	0.0024	0.0031	0.0561	1.4030	0.9983	0.1852	0.5612
	H_2_S	0.0020	0.0029	0.0540	1.3491	0.9983	0.1781	0.5396
	NH_3_	0.0022	0.0027	0.0520	1.3005	0.9985	0.1717	0.5202

The richness of studies performed so far in terms of number of sensors, gases investigated, and models employed is compared with our work in Table 3. Employing supervised machine learning with just one sensor, we attained stable classification and regression of three target gas species, which are independently grouped and well distinguished in the PCA space. These results confirm that PCA-based pretreatment allows for good visualization in a low-dimensional space and supports simultaneous gas identification and concentration prediction, providing a feasible and efficient ML-based method for gas sensing.

**Table 3:** Comparative evaluation with recent state-of-the-art reports^a^

Number of sensors	Target gases	Gas mixture	Gas concentration (ppm)	ML models used	Model accuracy (%)	Ref.
5 (commercialized MOS sensors)	2 (CO and CH_4_)	Binary	200	PCA, ICA, KPCA, KNN, MVRVM	98.33	34
1 (rGO/CuCoO_*x*_)	2 (NH_3_ and NO_2_)	Binary	0.05	PCA, DT, LDA, NB, SVM, KNN, EBT	98.1	42
8 (bare, cu, Pt, Ag-(TiO_2_, and ZnO))	5 (NO_2_, SO_2_, H_2_, O_2_, and ethanol)	Binary	0.1	PCA, DT, SVM, NB, KNN	100	48
3 (ZnO, NiO, and CuO)	4 (acetone, toluene, ethanol, and chloroform)	Binary and ternary	500	PCA, LR, KNN, NB, RF, LDA, ANN	99.81	53
1 (MgSb_2_O_6_)	3 (isoprene, *n*-propanol, and acetone)	Binary and ternary	0.1	SVM, GBDT, RF, KNN	98.94	54
4 (commercialised MOS sensors)	2 (CO and NO_2_)	Binary	10	CNN, BPNN	100	55
1 (rGO/In_2_O_3_)	5 (NH_3_, CO, H_2_S, NO_2,_ and SO_2_)	Binary	0.1	PCA, KNN, LR, RF, DT, NB	99.97	This work

a PCA: principal component analysis, SVM: support vector machine, GBDT: gradient boosting decision tree, RF: random forest, KNN: K-nearest neighbor, DT: decision tree, EB: ensembled bagged trees, LDA: linear discriminant analysis, LR: logistic regression, NB: Naïve Bayes, BPNN: back-propagation neural network, CNN: convolutional neural network, ICA: independent component analysis, KPCA: Kernel principal component analysis, MVRVM: multivariate relevance vector machine.

## 4. Conclusion

This research reports the fabrication of rGO/In_2_O_3_ nanocomposite gas sensors for predictive sensing in complex mixed-gas environments. Optimizing the rGO concentration systematically enhanced the oxygen vacancy density, while interfacial charge transfer assisted in transcending metal oxide sensors' inherent limitations of low sensitivity at sub-ppm levels and lack of selectivity. Building on the optimized sensor, we developed a machine learning framework that performed exceptionally well on classification and regression tasks. Individual gas clusters were resolved with 99.7% accuracy, and the regression model repeatedly predicted concentrations of previously untrained target gases. Collectively, these findings demonstrate the potential of a single rGO/In_2_O_3_ sensor, augmented by data-driven analysis, to provide ultra-sensitive, selective, and predictive toxic gas detection. The findings further open avenues toward practical and energy-efficient gas sensing platforms suitable for real-world environmental and health monitoring applications.

## Conflicts of interest

The authors declare no conflict of interest.

## Supplementary Material

NA-008-D5NA01092F-s001

## Data Availability

The data sets supporting this article are not publicly available at the time of publication as they are not currently in a format suitable for broad distribution or reuse. However, the data can be provided by the authors upon reasonable request. Supplementary information (SI): detailed structural, optical, and PL characterization (XRD W–H analysis, UV-vis spectra, Tauc plots, PL deconvolution), comprehensive gas sensing performance data under single and mixed gas conditions, repeatability and long-term stability studies, and complete machine learning analysis including feature extraction, correlation matrices, PCA, feature loadings, and classification results. See DOI: https://doi.org/10.1039/d5na01092f.
