# Supplementary material for: Predictive mixed-gas detection using rGO/In2O3 nanocomposite sensors assisted by machine learning
Source: Nanoscale Adv. 2026 Feb 2;8(6):1958–74. doi: 10.1039/d5na01092f (PMC12915307; doi:10.1039/d5na01092f)
Supplement: NA-008-D5NA01092F-s001 [file NA-008-D5NA01092F-s001.pdf]

Supplementary Information (SI) for Nanoscale Advances.
This journal is © The Royal Society of Chemistry 2026

Supporting Information

Predictive Mixed-Gas Detection Using rGO/ In_2O_3 Nanocomposite Sensors Assisted by Machine Learning

Tanya Sood[1], Saikat Chattopadhyay[2], P. Poornesh[1*]

[1]Manipal Institute of Technology, Manipal Academy of Higher Education, Manipal, India

[2]Department of Physics, School of Basic Sciences, Manipal University Jaipur, Jaipur 303007, India

[*]Corresponding author: poornesh.p@manipal.edu, poorneshp@gmail.com

1. Structural insights from XRD

Among the various techniques used to account for both crystallite size and lattice strain in the broadening of XRD peaks, the Williamson-Hall (W-H) approach stands out for its simplicity and ease of application. This method assumes that the observed peak broadening (β) arises from two main contributions: one due to finite crystallite size (β_D) and the other from microstrain within the crystal lattice (β_ε), as described by the equation [1]:

$$\beta = \beta_D + \beta_\varepsilon \quad \text{(S1)}$$

The strain-related broadening component is modeled using the relation:

$$\beta_\varepsilon = 4\varepsilon \tan\theta$$

(S2)

By incorporating both effects, the total broadening is given by:

$$\beta = \frac{K\lambda}{D \cos\theta} + 4\varepsilon \tan\theta$$

(S3)

This can be rearranged into a linear form:

$$\beta \cos\theta = \frac{K\lambda}{D} + 4\varepsilon \sin\theta$$

(S4)

In this method, a plot is constructed with 4 sinθ on the x-axis and β cosθ on the y-axis. From the linear fit of this plot, the slope corresponds to the microstrain (ε), and the y-intercept allows estimation of the average crystallite size (D) of the rGO-based samples. Figure 1 shows the W-H plot for rIO0 and rGO-based samples.

For calculating dislocation density (δ):

$$\delta = \frac{1}{D^2} \quad \text{(S5)}$$

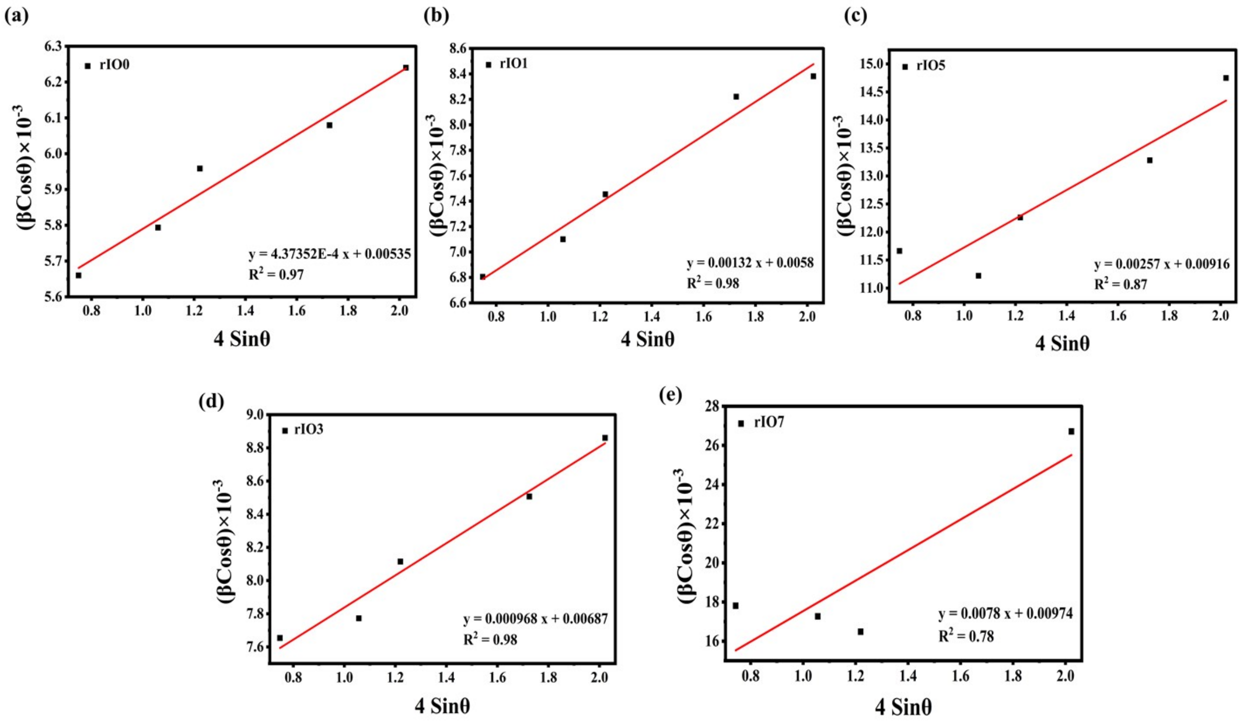

Figure 1. W-H Plot of rIO0 and rGO/ In_2O_3 composites.

2. UV-Vis Spectroscopy

Examining the optical properties of semiconducting materials is essential as it provides insights into their band structures and the nature of electronic transitions. Figure 2 (a) shows the optical transmittance spectra of pure In_2O_3 and rGO/ In_2O_3 nanocomposites over the spectral range of 300–1100 nm. The pure In_2O_3 nanostructures exhibit excellent transparency, with transmittance reaching approximately 92%. The oscillations observed in the 400–1100 nm range suggest the presence of smooth film interfaces. However, the transmittance decreases with increasing rGO content. For instance, in the rIO7 sample, the transmittance drops to 63%. This reduction is attributed to the strong optical absorption exhibited by rGO across the UV-

visible region. This absorption originates from the restoration of the sp^2 carbon network during the reduction of graphene oxide. As GO is reduced to rGO, oxygen-containing functional groups are partially removed, leading to the reformation of the conjugated π-electron system. The resulting extended delocalized electron networks enable efficient light absorption over a broad spectral range [2]. Moreover, the transmittance oscillations observed in pure In_2O_3 disappear in the rGO composite samples. This is due to the introduction of surface irregularities by rGO, which disrupts interfacial coherence and leads to incoherent light scattering [3].

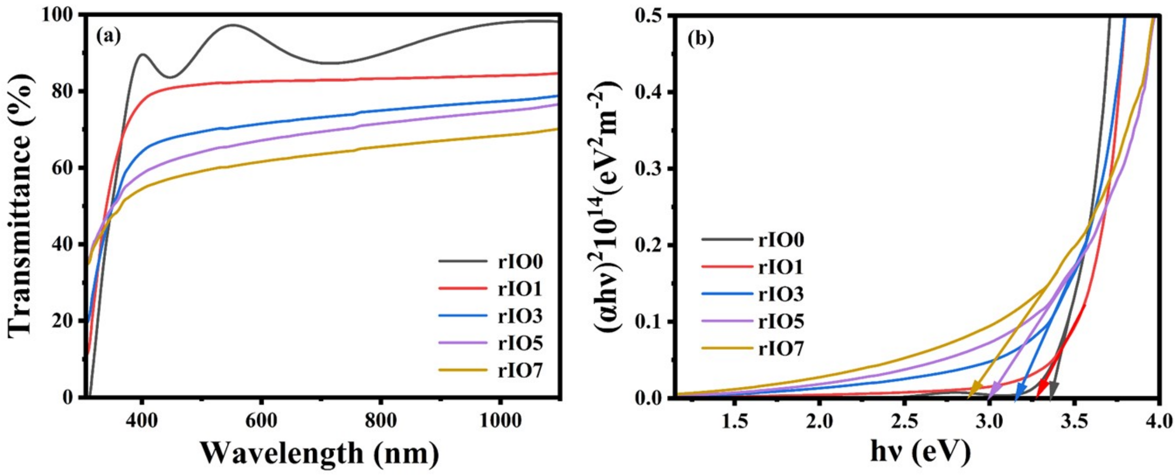

Figure 2. (a) Transmittance spectra and (b) Tauc's plot of In_2O_3 and rGO/ In_2O_3 nanocomposites.

Tauc's relation is utilized to determine the energy bandgap (E_g) values, expressed as [4],

$$(\alpha h\nu)^n = B\,(h\nu - E_g) \tag{S4}$$

In the equation, α denotes the absorption coefficient, hν denotes photon energy, E_g indicates the optical bandgap, while B is a constant that depends on the transition probability. The exponent n defines the type of the transition. In Tauc's plot, $h\nu$ is plotted on x-axis and $(\alpha h\nu)^{1/n}$ is plotted on the y-axis. For direct allowed transitions, n = ½, while for indirect allowed transitions, n = 2. The absorption coefficient (α) can be determined utilizing the Beer-Lamberts law [4]

$$\alpha = \frac{1}{t}\ln\left(\frac{1}{T}\right) \tag{S5}$$

Where t denotes the obtained film's thickness and T represents the transmittance obtained by the films. The optical band gap, E_g is derived from the intersect of the straight line with the hν axis of Tauc's plot. As shown in the Table 1, the band gap value for pure In_2O_3 is found to be

3.36 eV, which aligns well with values reported in the literature. Upon increasing rGO concentration from 1 to 7 wt%, the band gap decreases progressively from 3.28 eV to 2.89 eV. This reduction can be attributed to multiple factors. Firstly, the restoration of the sp^2 carbon network in rGO leads to the formation of extended delocalized π-electron systems, which can interact with the conduction band of In_2O_3 [4]. Secondly, the formation of rGO/ In_2O_3 heterojunctions introduces interfacial electronic states, which alter the electronic structure and reduce the overall band gap. Additionally, the improved planar structure of rGO facilitates more efficient charge carrier transport, further contributing to the narrowing of the band gap[5].

Table 1 Band Gap (E_g) of In_2O_3 and rGO/ In_2O_3 nanocomposites

rGO concentration (wt %)	E_g (eV)
0	3.36
1	3.28
3	3.16
5	2.99
7	2.89

3. Photoluminescence spectroscopy

The photoluminescence (PL) spectroscopy analysis, conducted with an excitation wavelength of 335 nm, reveals presence of defects in In_2O_3 and rGO/ In_2O_3 nanocomposite thin films. Indium oxide thin films typically exhibit intrinsic defects such as antisites, vacancies, and interstitials of indium as well as oxygen. The PL spectra of In_2O_3 and rGO/ In_2O_3 nanocomposite thin films, shown in Figure 3, were analysed through Gaussian deconvolution, offering comprehensive details about the defect states responsible for the detected PL emissions. This method enables the precise identification and characterization of various defects by isolating the contributions of distinct emission centers.

For In_2O_3 thin films, the photoluminescence (PL) spectra reveal emissions that can be broadly categorized into a prominent ultraviolet (UV) or near-band-edge (NBE) emission band, along with significant deep-level emissions (DLEs). The NBE emission typically corresponds to higher energy transitions near the band gap, while the DLEs arise from energies associated with defect states within the band gap. The ultraviolet emission, with an energy of 3.15 eV, is attributed to the recombination of free excitons, which may occur via exciton-exciton collisions. The near-band-edge (NBE) emission peak (3.15 eV) appeared weaker when

compared to some of the peaks from deep-level emission. Such behaviour suggests that the relaxation of photoexcited carriers from the conduction band to defect states occurs more rapidly than their recombination with valence band holes, so the contribution of quantum confinement effect to the observed photoluminescence can be ruled out [6]. On the other hand, DLE bands, observed at energies of 2.25 eV-2.89 eV, are generally linked to intrinsic point defects, including oxygen vacancies, interstitials, or antisites, within the In_2O_3 films [7]. These visible emissions are believed to result from the recombination of deeply trapped electrons with delocalized holes in the valence band or from delocalized electrons recombining with deeply trapped holes in the conduction band. The violet-blue emission peaks observed around 2.89 eV, and 2.63 eV could be attributed to singly ionized oxygen vacancy (V_o^+) defects and deeper oxygen-vacancy related defects [8]. The green emission peak observed around 2.25 eV is related to complex defect states involving indium vacancy (V'''_{in}) defects or oxygen vacancy-indium vacancy pairs ($V_o - V_{in}$) [8].

Figure 3 illustrates how different rGO composition influences the position and intensity of defect-relates peaks. The red-shift of both NBE and DL emission peaks is observed for all rGO compositions except 5 wt%. This red shift is due to the electronic coupling between rGO and In_2O_3. Further, for 5 wt%, blue shift of these peaks is observed which is attributed to the Moss-Burstein effect caused by the increased electron density from oxygen vacancies [9]. Furthermore, the PL intensity is maximum for 5 wt%, which is attributed to the formation of optimal p-n heterojunctions which supplies mobile electrons and adsorption sites, heightening chemiresistive response.

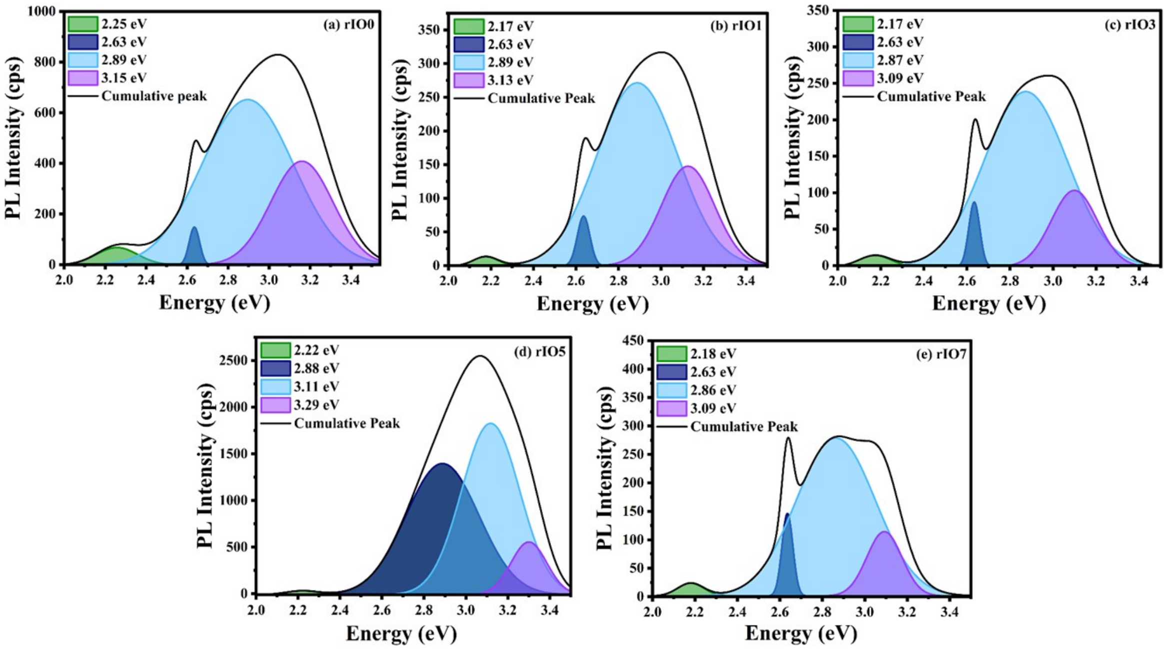

Figure 3. Gaussian Deconvoluted PL emission spectra of In_2O_3 and rGO/ In_2O_3 nanocomposites.

4. Gas Sensing Measurements:

4.1. Operating Temperature Studies:

The operating temperature plays a crucial role in determining the sensing performance of metal oxide semiconductor (MOS) gas sensors, as it directly governs gas adsorption, desorption, and surface reaction kinetics. To determine the optimal operating temperature, both rIO0 and rIO5 sensors were systematically evaluated for their response to 4 ppm H_2S over a temperature range of 150-350 °C, as shown in Figure 4. The sensor response increases progressively with temperature from 150 °C to 250 °C due to enhanced activation of surface-adsorbed oxygen species and improved reaction kinetics with H_2S molecules. At temperatures below 250 °C, the thermal energy is insufficient to overcome the activation barrier required for effective gas-surface interactions, resulting in lower sensor response. Beyond 250 °C, the response decreases because elevated temperatures promote rapid desorption of gas molecules from the sensor surface, reducing the time required for surface reactions [10]. Therefore, 250 °C represents an optimal balance between adsorption and reaction kinetics, yielding the maximum sensor response. Accordingly, all subsequent gas sensing measurements were performed at this temperature.

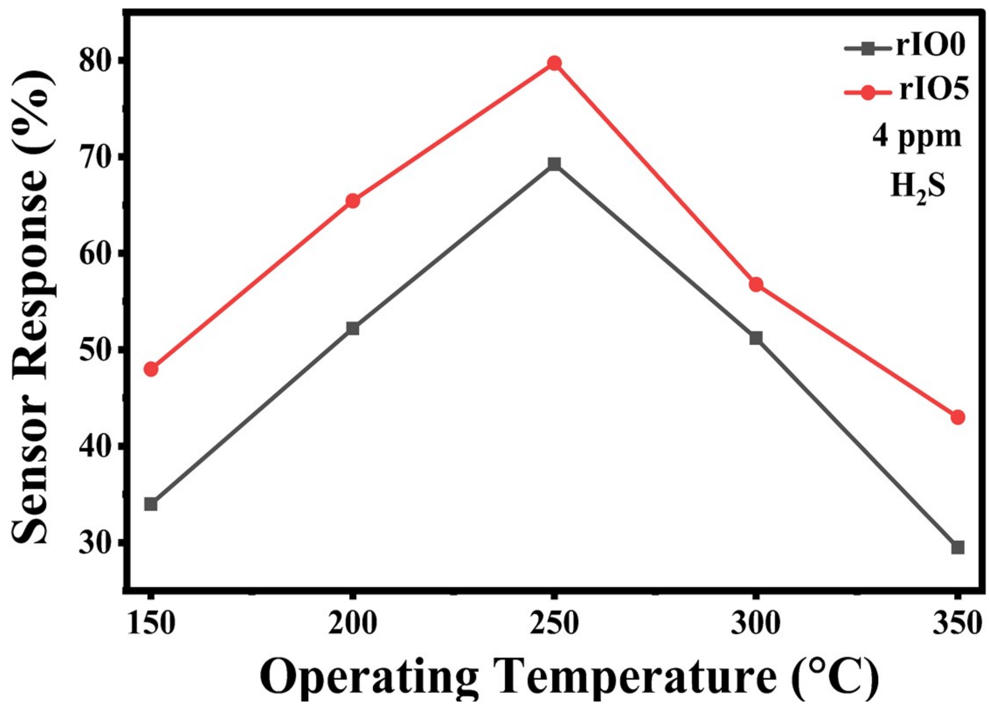

Figure 4. Operating temperature-dependent sensing response of nanostructured rIO0 and rIO5 sensors toward 4 ppm H_2S, illustrating the optimization of the operating temperature.

4.2. Sensing Parameters of rIO0 and rGO/ In_2O_3 Sensors at Different H_2S Concentrations (250 °C)

Table 2 Sensor Response values for different concentrations of H_2S gas for rIO0 and rGO/ In_2O_3 sensors.

Gas Concentration (ppm)	Sensor Response (%)				
	**rIO0**	**rIO1**	**rIO3**	**rIO5**	**rIO7**
**0.1**	7.4 ± 1.8	9.6 ± 1.8	8.9 ± 5.3	7.9 ± 0.5	6.8 ± 1.1
**0.5**	29.4 ± 1.9	29.9 ± 1.2	36.1 ± 1.5	39.2 ± 1.1	25.9 ± 0.9
**1**	39.3 ± 1.3	41.6 ± 1.0	41.6 ± 1.1	43.3 ± 0.3	35.5 ± 1.1
**2**	50.1 ± 0.8	54.2 ±1.1	54.8 ± 0.9	49.8 ± 0.5	47.4 ± 0.9
**3**	58.6 ± 1.1	61.9 ± 1.0	62.8 ± 0.7	57.7 ± 0.4	55.8 ± 0.8
**4**	69.2 ± 0.5	71.4 ± 0.6	71.7 ± 0.9	79.7 ± 0.3	67.9 ± 1.1

Table 3 Response Time values for different concentrations of H_2S gas for rIO0 and rGO/ In_2O_3 sensors.

Gas Concentration (ppm)	Response Time (s)				
	rIO0	rIO1	rIO3	rIO5	rIO7
**0.1**	57 ± 2	48 ± 2	35 ± 2	46 ± 5	94 ± 1
**0.5**	87 ± 5	90 ± 14	96 ± 4	77 ± 9	131 ± 8
**1**	136 ± 2	72 ± 2	53 ± 1	75 ± 9	126 ± 4
**2**	39 ± 4	57 ± 1	45 ± 1	72 ± 7	111 ± 1
**3**	61 ± 9	60 ± 1	42 ± 1	97 ± 8	129 ± 2
**4**	75 ± 4	83 ± 6	49 ± 2	59 ± 7	158 ± 10

Table 4 Recovery Time values for different concentrations of H_2S gas for rIO0 and rGO/ In_2O_3 sensors.

Gas Concentration (ppm)	Recovery Time (s)				
	rIO0	rIO1	rIO3	rIO5	rIO7
**0.1**	113 ± 6	144 ± 10	127 ± 6	96 ± 4	109 ± 14
**0.5**	147 ± 13	102 ± 3	128 ± 5	124 ± 8	139 ± 3
**1**	139 ± 13	123 ± 4	122 ± 9	129 ± 10	126 ± 6
**2**	135 ± 5	115 ± 6	123 ± 5	128 ± 9	130 ± 8
**3**	146 ± 17	114 ± 6	122 ± 5	136 ± 7	130 ± 5
**4**	131 ± 11	120 ± 2	119 ± 4	202 ± 12	143 ± 5

4.3. Repeatability and long-term stability measurements:

The stability of the rIO5 sensor was evaluated through both repeatability and long-term stability tests. As shown in Figure 4 (a), repeatability measurements were performed by repeatedly exposing the sensor to 4 ppm H_2S at the optimal operating temperature of 250 °C. The sensor response exhibited no significant variation over successive cycles, demonstrating excellent repeatability.

In addition, long-term stability was assessed by measuring the sensor response to 4 ppm H_2S at intervals of 5 days. As illustrated in Figure 4 (b), only minor fluctuations in response were observed over time, indicating good long-term stability of the nanocomposite sensor. These results confirm the reliability and robustness of the rIO5 sensor for sustained gas sensing applications.

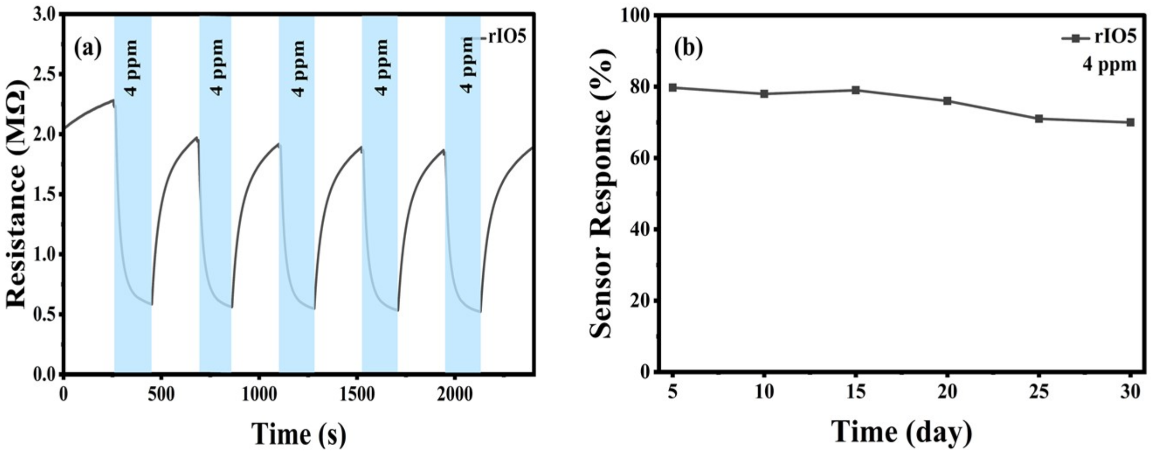

Figure 5. (a) Repeatability and (b) long-term stability of the rIO5 sensor measured toward 4 ppm H_2S at the optimal operating temperature of 250 °C.

4.4. Sensing Parameters of rIO5 Sensor in mixed-gas environment (250 °C).

Table 5 Sensor response of rIO5 toward different concentrations of H_2S in the presence of interfering gases.

Gas Concentration (ppm)	Sensor Response (%)				
	H_2S alone	H_2S + CO	H_2S + NH_3	H_2S + SO_2	H_2S + NO_2
**0.1**	7.9	25	31.8	4.3	36.5
**0.5**	39.2	52	45.1	16.7	62.7
**1**	43.3	66.1	50	29.2	76.8
**2**	49.8	78.8	55.5	40	84
**3**	57.7	80.4	57.3	37	87.1
**4**	79.7	90.6	88.9	76.8	86.7

Table 6 Response Time of rIO5 toward different concentrations of H_2S in the presence of interfering gases.

Gas Concentration (ppm)	Response Time (s)				
	H_2S alone	H_2S + CO	H_2S + NH_3	H_2S + SO_2	H_2S + NO_2
**0.1**	46 ± 5	181 ± 5	234 ± 3	190 ± 5	213 ± 5
**0.5**	77 ± 9	82 ± 2	124 ± 5	97 ± 11	93 ± 4
**1**	75 ± 9	96 ± 2	59 ± 3	204 ± 3	113 ± 3
**2**	72 ± 7	81 ± 6	65 ± 5	103 ± 3	76 ± 2
**3**	97 ± 8	98 ± 8	78 ± 4	118 ± 3	35 ± 3
**4**	59 ± 7	50 ± 1	278 ± 15	215 ± 6	152 ± 6

Table 7 Recovery time of rIO5 toward different concentrations of H_2S in the presence of interfering gases.

Gas Concentration (ppm)	Recovery Time (s)				
	H_2S alone	H_2S + CO	H_2S + NH_3	H_2S + SO_2	H_2S + NO_2
**0.1**	96 ± 4	252 ± 8	189 ± 4	165 ± 5	223 ± 6
**0.5**	124 ± 8	234 ± 12	193 ± 3	225 ± 7	211 ± 4
**1**	129 ± 10	271 ± 2	170 ± 5	210 ± 8	255 ± 6
**2**	128 ± 9	267 ± 5	197 ± 6	198 ± 8	241 ± 4
**3**	136 ± 7	243 ± 4	176 ± 7	184 ± 7	216 ± 5
**4**	202 ± 12	242 ± 8	298 ± 9	214 ± 5	177 ± 3

Table 8 Sensor response of rIO5 toward different concentrations of CO in the presence of interfering gases.

Gas Concentration (ppm)	Sensor Response (%)				
	CO alone	CO + H_2S	CO + NH_3	CO + SO_2	CO + NO_2
**0.5**	20.4	7.4	14.1	11.5	13.3
**1**	30.1	23.1	25.3	19.2	20
**2**	42.6	36.5	35.2	28.9	26.7
**3**	48	44.4	43.3	37.9	40

**4**	58	57.1	54.9	47.3	46.7

Table 9 Response Time of rIO5 toward different concentrations of CO in the presence of interfering gases.

**Gas Concentration (ppm)**	**Response Time (s)**				
	**CO alone**	**CO + H_2S**	**CO + NH_3**	**CO + SO_2**	**CO + NO_2**
**0.5**	195 ± 9	44 ± 4	163 ± 10	186 ± 2	216 ± 6
**1**	155 ± 2	54 ± 3	162 ± 4	182 ± 2	193 ± 1
**2**	141 ± 3	96 ± 1	143 ± 3	194 ± 4	219 ± 2
**3**	142 ± 5	130 ± 1	175 ± 5	223 ± 4	226 ± 9
**4**	167 ± 4	190 ± 2	210 ± 6	222 ± 5	272 ± 4

Table 10 Recovery Time of rIO5 toward different concentrations of CO in the presence of interfering gases.

**Gas Concentration (ppm)**	**Recovery Time (s)**				
	**CO alone**	**CO + H_2S**	**CO + NH_3**	**CO + SO_2**	**CO + NO_2**
**0.5**	164 ± 3	254 ± 13	180 ± 3	174 ± 7	199 ± 12
**1**	127 ± 2	154 ± 15	171 ± 7	196 ± 4	198 ± 2
**2**	138 ± 4	209 ± 9	182 ± 4	199 ± 4	199 ± 3
**3**	140 ± 3	174 ± 8	179 ± 2	183 ± 6	210 ± 6
**4**	180 ± 7	203 ± 6	190 ± 3	214 ± 4	221 ± 6

Table 11 Sensor response of rIO5 toward different concentrations of NH_3 in the presence of interfering gases.

**Gas Concentration (ppm)**	**Sensor Response (%)**				
	**NH_3 alone**	**NH_3 + H_2S**	**NH_3 + CO**	**NH_3 + SO_2**	**NH_3 + NO_2**
**0.5**	18.3	21.6	13.7	12.6	11.8

**1**	32.2	30.4	22.3	20.3	17.9
**2**	40.5	42.1	34.2	31.6	26.5
**3**	48.1	48.2	41.5	39.1	31.8
**4**	58.6	58.9	48.9	45.9	40.3

Table 12 Response Time of rIO5 toward different concentrations of NH_3 in the presence of interfering gases.

**Gas Concentration (ppm)**	**Response Time (s)**				
	**NH_3 alone**	**$NH_3 + H_2S$**	**NH_3 + CO**	**$NH_3 + SO_2$**	**$NH_3 + NO_2$**
**0.5**	141 ± 3	203 ± 6	175 ± 6	203 ± 7	233 ± 4
**1**	163 ± 3	202 ± 3	209 ± 3	171 ± 5	193 ± 2
**2**	121 ± 2	170 ± 2	195 ± 2	198 ± 4	213 ± 9
**3**	114 ± 3	188 ± 4	200 ± 8	210 ± 5	233 ± 4
**4**	120 ± 2	199 ± 5	233 ± 6	217 ± 3	245 ± 4

Table 13 Recovery Time of rIO5 toward different concentrations of NH_3 in the presence of interfering gases.

**Gas Concentration (ppm)**	**Recovery Time (s)**				
	**NH_3 alone**	**$NH_3 + H_2S$**	**NH_3 + CO**	**$NH_3 + SO_2$**	**$NH_3 + NO_2$**
**0.5**	145 ± 3	189 ± 2	190 ± 4	175 ± 4	255 ± 13
**1**	148 ± 2	149 ± 2	202 ± 4	188 ± 3	201 ± 3
**2**	130 ± 3	210 ± 1	221 ± 5	211 ± 1	234 ± 8
**3**	119 ± 3	177 ± 2	190 ± 1	209 ± 2	213 ± 7
**4**	139 ± 4	225 ± 5	199 ± 2	228 ± 3	236 ± 4

4.5. Classification and regression analysis of gases using machine learning:

Features Used:

Table 14 Definition of features extracted from dynamic gas sensing response curves for machine learning analysis.

Feature No.	Feature Name	Definition/ Description
1	Time	Time index corresponding to each resistance data point during gas exposure and recovery, representing the temporal evolution of the sensor response.
2	Resistance	Instantaneous electrical resistance of the sensor measured at each time step during gas exposure and recovery.
3	Constant gas ppm	Concentration of the interfering gas maintained constant during mixed-gas sensing experiments.
4	Target gas ppm	Concentration of the target gas introduced during sensing, used for both classification and regression tasks.
5	Constant gas type	Identity of the interfering gas (e.g., NH_3, CO, NO_2, SO_2) present during mixed-gas measurements.
6	Target gas type	Identity of the target gas (H_2S, NH_3, or CO) whose concentration and presence are to be predicted.
7	Response Time	Time required for the sensor resistance to reach 90% of the maximum response upon exposure to the target gas.
8	Recovery Time	Time required for the sensor resistance to return to 90% of its baseline value after removal of the target gas.
9	Sensor Response	Relative change in sensor resistance upon gas exposure, calculated as $S = (R_a - R_g)/R_a$ for reducing gases and $S = (R_g - R_a)/R_a$ for oxidizing, representing response magnitude.

The correlation matrices of the processed datasets are illustrated in Figure 6. To enable quantitative analysis, categorical gas types were converted into numerical form using label encoding. For the target gas dataset, the gases were mapped as CO = 0, H_2S = 1, and NH_3 = 2. In the case of the background (mixing) gases, the encoding was extended to include CO = 0, H_2S = 1, NH_3 = 2, NO_2 = 3, and SO_2 = 4. Furthermore, the kinetic parameters obtained from the experiments were stored in a structured format, where the primary values were clearly separated from their corresponding uncertainties. Specifically, the response time and recovery time were stored as Responsetime_value and Recoverytime_value, respectively. This systematic representation ensures that both the measured values and their uncertainties are preserved, providing a reliable foundation for statistical evaluation and correlation analysis.

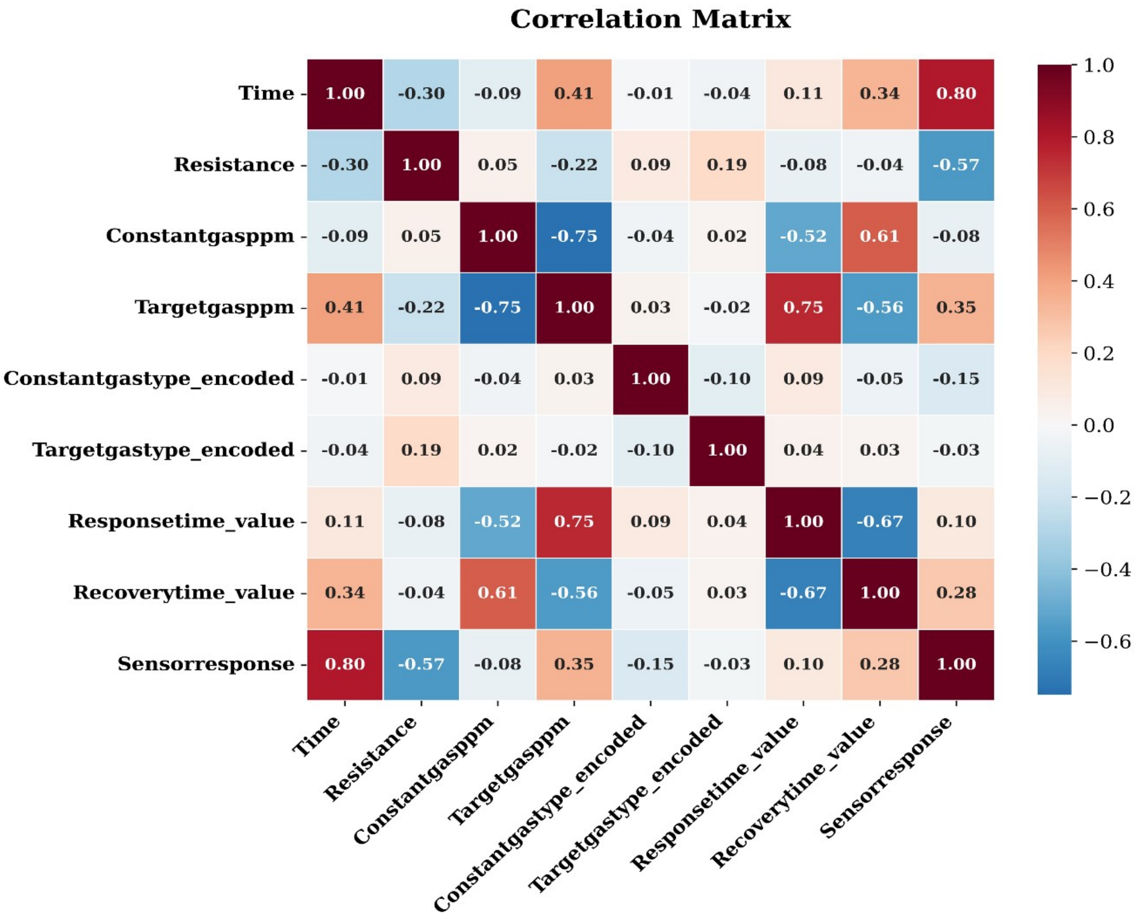

Figure 6. Correlation matrix of the nine extracted features from sensor response curves, used to evaluate interdependence prior to PCA.

The Figure 7 illustrates the proportion of total variance in the dataset explained by the first five principal components (PCs). In Figure 7 (a), the variance explained by each individual component is presented. The first principal component (PC1) alone accounts for 37.9% of the

total variance, indicating that more than one-third of the overall variation is concentrated along a single axis. The second principal component (PC2) contributes an additional 28.9%, such that together PC1 and PC2 capture 66.8% of the total variance. The subsequent components-PC3 (12.6%), PC4 (9.6%), and PC5 (6.0%)-progressively explain smaller portions of the remaining variance. These results highlight the dominant role of PC1 and PC2, while the later components provide comparatively minor contributions.

Figure 7 (b) presents the cumulative variance explained by successive inclusion of the components. Starting with 37.9% for PC1, the cumulative variance increases to 66.8% with the addition of PC2, 79.4% with PC3, 88.9% with PC4, and reaches 94.9% with PC5. The curve reflects diminishing returns beyond the first two components, as each additional PC contributes smaller increments. Overall, projecting the original eleven features onto the first five principal components retains 94.9% of the dataset's information while reducing the dimensionality to five. This dimensionality reduction captures the essential data structure with minimal information loss, thereby simplifying subsequent analysis without compromising representational integrity.

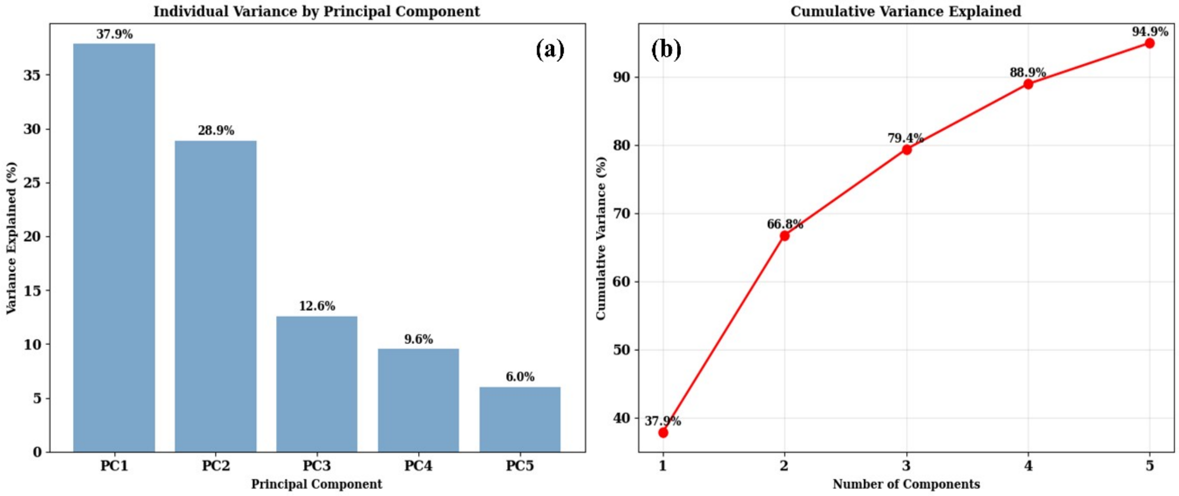

Figure 7. PCA analysis of the dataset: (a) Variance contribution of individual principal components and (b) cumulative variance explained. The first two and first three components account for 66.8% and 79.4% of the total variance, respectively.

The feature loading analysis shown in Figure 9, highlights the contribution of individual variables to each principal component. For PC1, which explains 37.9% of the variance, the largest positive loadings are associated with response time (0.425), and target gas concentration (0.434), while recovery time (-0.399) exhibit strong negative loadings. This distribution

suggests that PC1 primarily reflects a contrast between sensors that exhibit faster responses at higher concentrations, even though with greater uncertainty, and those that demonstrate slower recovery dynamics. PC2, accounting for 28.9% of the variance, is dominated by positive contributions from sensor response (0.591) and measurement time (0.554), opposed by negative contributions from resistance (-0.402) and, to a smaller extent, constant gas concentration (-0.124). This indicates that PC2 differentiates between sensors producing stronger response signals over longer measurement durations and those characterized by higher baseline resistance. Similarly, for PC3. This component primarily distinguishes among different gas species rather than sensor kinetics, capturing categorical variability associated with gas identity. Overall, the first three principal components summarize the most significant patterns: PC1 reflects the trade-off between rapid, high-concentration responses and slower recovery behavior, PC2 represents the relationship between signal intensity, measurement duration, and baseline resistance, and PC3 separates data based on gas type.

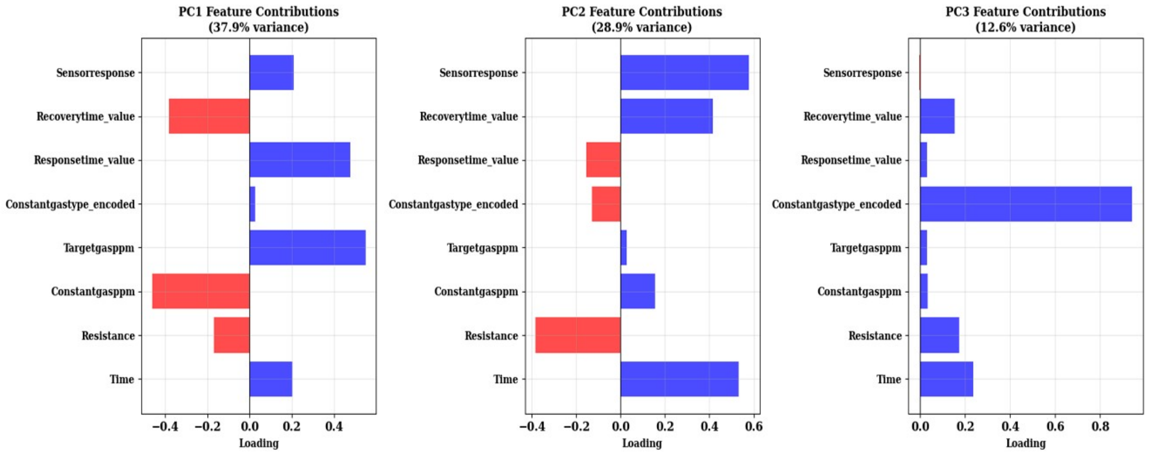

Figure 9. Feature loadings.

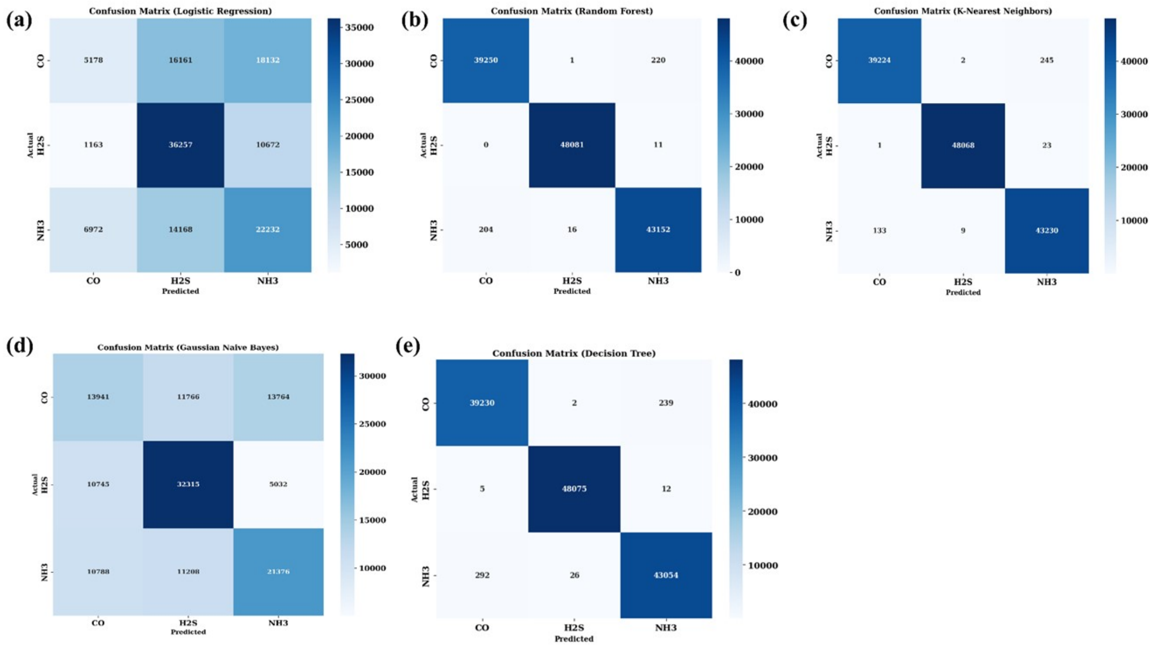

Figure 10. Confusion matrices of different supervised ML models.

References

[1] Aparna C, Mahesha MG, Kumara Shetty P. Structural and optical properties of indium oxide thin films synthesized at different deposition parameters by spray pyrolysis. Mater Today Proc 2022;55:141–7. https://doi.org/10.1016/j.matpr.2022.01.048.

[2] Wang S, Dong Y, He C, Gao Y, Jia N, Chen Z, et al. The role of sp2/sp3 hybrid carbon regulation in the nonlinear optical properties of graphene oxide materials. RSC Adv 2017;7:53643–52. https://doi.org/10.1039/c7ra10505c.

[3] Tene T, Guevara M, Benalcázar Palacios F, Morocho Barrionuevo TP, Vacacela Gomez C, Bellucci S. Optical properties of graphene oxide. Front Chem 2023;11. https://doi.org/10.3389/fchem.2023.1214072.

[4] Gupta S, Narajczyk M, Sawczak M, Bogdanowicz R. Perspectives on electron transfer kinetics across graphene-family nanomaterials and interplay of electronic structure with defects and quantum capacitance. Sci Rep 2025;15. https://doi.org/10.1038/s41598-025-04357-x.

[5] Tian H, Wan C, Xue X, Hu X, Wang X. Effective electron transfer pathway of the ternary TiO2/RGO/Ag nanocomposite with enhanced photocatalytic activity under visible light. Catalysts 2017;7. https://doi.org/10.3390/catal7050156.

[6] Beena D, Lethy KJ, Vinodkumar R, Detty AP, Pillai VPM, Ganesan V. Photoluminescence in laser ablated nanostructured indium oxide thin films 2010;489:215–23. https://doi.org/10.1016/j.jallcom.2009.09.055.

[7] Anand K, Kaur J, Singh RC, Thangaraj R. Structural, optical and gas sensing properties of pure and Mn-doped In2O3 nanoparticles. Ceram Int 2016;42:10957–66. https://doi.org/10.1016/j.ceramint.2016.03.233.

[8] Arooj S, Xu T, Hou X, Wang Y, Tong J, Chu R, et al. Green emission of indium oxide: Via hydrogen treatment. RSC Adv 2018;8:11828–33. https://doi.org/10.1039/c8ra00654g.

[9] Khurshid F, Jeyavelan M, Hudson MSL, Nagarajan S. Ag-doped ZnO nanorods embedded reduced graphene oxide nanocomposite for photo-electrochemical applications. R Soc Open Sci 2019;6. https://doi.org/10.1098/rsos.181764.

[10] Shao X, Zhang D, Tang M, Zhang H, Wang Z, Jia P, et al. Amorphous Ag catalytic layer-SnO2 sensitive layer-graphite carbon nitride electron supply layer synergy-enhanced hydrogen gas sensor. Chemical Engineering Journal 2024;495. https://doi.org/10.1016/j.cej.2024.153676.
